# Supplementary material for: The plasticity of the pyruvate dehydrogenase complex confers a labile structure that is associated with its catalytic activity
Source: PLoS One. 2020 Dec 28;15(12):e0243489. doi: 10.1371/journal.pone.0243489 (PMC7769281; doi:10.1371/journal.pone.0243489)
Supplement: S1 File — (PDF) [file pone.0243489.s001.pdf]

S1 Fig.

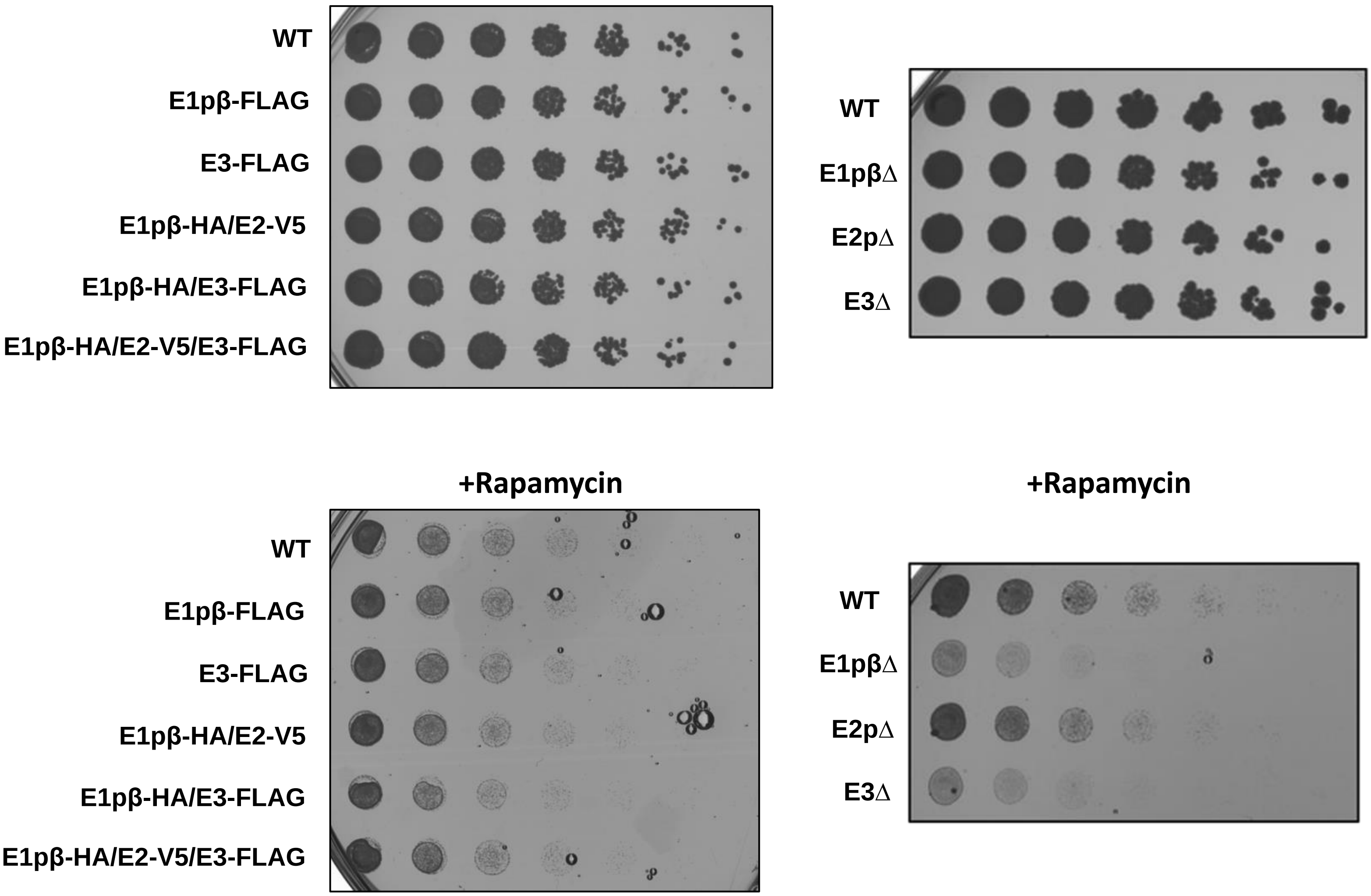

S1 Fig. The epitope-tagging of PDC subunits did not affect the growth and cellular function of the strains. Spotting assay of WT (BY4741), E1pβ-FLAG (Pdb1-5xFLAG), E3-FLAG (Lpd1-5xFLAG), E1pβ-HA/E2p-V5 (Pdb1-3xHA/Lat1-V5), E2p-V5/E3-FLAG (Lat1-V5/Lpd1-5xFLAG), and E1pβ-HA/E2p-V5/E3-FLAG (Pdb1-3xHA/Lat1-V5/Lpd1-5xFLAG) on YPD plates and rapamycin (10 nM) containing YPD plates along with E1pβΔ (*pdb1Δ*), E2Δ (*lat1Δ*), and E3Δ (*lpd1Δ*).

S2 Fig.

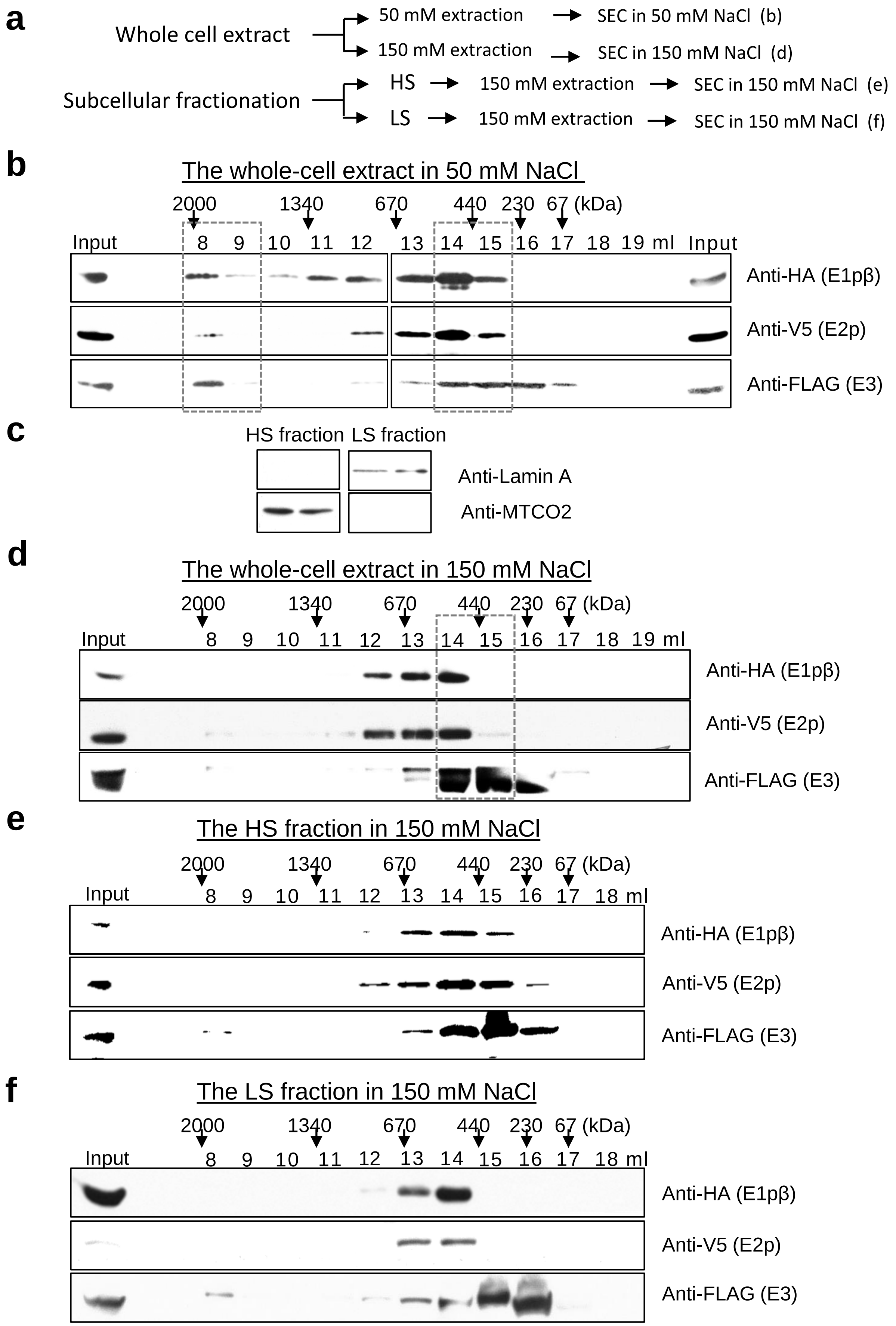

S2 Fig. The SEC profiles of yeast extract display sub-megadalton PDC components. (a) Flowchart of SEC experiments. (b) Size fraction profiles of yPDC components from whole-cell extract in a buffer containing 50 mM NaCl. (c) Detection of standard proteins, Lamin A for nuclei and MTCO2 for mitochondria as a subcellular fractionation control in mitochondria-enriched HS and nuclei-enriched LS fractions from two biological replicates. For (d)-(f), Size fraction profiles of yPDC components of Pdb1-3xHA/Lat1-V5/Lpd1-5xFLAG strain from (d) whole-cell extract in a buffer containing 150 mM NaCl, (e) the HS fraction in 150 mM NaCl, and (f) the LS fraction in 150 mM NaCl. Elution volumes in the SEC via Superose 6 column for every other 500 µl fraction are indicated with the expected size of eluted proteins based on standard proteins. Boxes in dashed lines highlight the peak fractions.

S3 Fig.

a

Detected Protein \ yPDC-AP bait	dNSAF	
	E1pβ-FLAG AP	E3-FLAG AP
E1pβ (Pdb1)	0.3258	0.009001
E1pα (Pda1)	0.2092	0.006754
E2p (Lat1)	0.02917	0.01263
E3 (Lpd1)	0.1130	0.3709
E3BP (Pdx1)	0.006775	0.02846

b

Detected Protein \ peak fraction for each AP	dNSAF	
	yE1pβ-AP SEC @ 14.5 ml	yE3-AP SEC @ 16 ml
yE1pβ (Pdb1)	0.3458	0.006628
yE1pα (Pda1)	0.3177	0.005217
yE2p (Lat1)	0.02249	0.0007306
yE3 (Lpd1)	0.0008900	0.6789
yE3BP (Pdx1)	0.0009750	0.004581

S3 Fig. Affinity purification of yPDC subunits suggests the lability of the complex

(a) Relative protein levels (dNSAF) of yPDC components found in MudPIT analyses of the FLAG purified yPDC via its subunits E1β (Pdb1) and E3 (Lpd1) from Pdb1-5xFLAG and Lpd1-5xFLAG, respectively. (b) Relative protein levels (dNSAF) of yPDC components found in MudPIT analyses of a peak fraction of SEC following E1β-AP and E3-AP in 350 mM NaCl condition at the indicated elution volume.

S4 Fig.

a

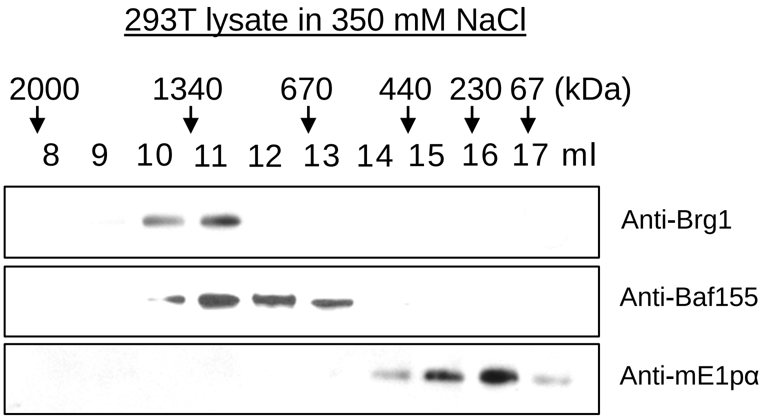

b

Complex	Size in 350 mM NaCl
mPDC E1pα/β	~230 kDa
yPDC E1pβ	~440 kDa
yPDC E3	~230 kDa
SAGA	1-2 MDa
SWI/SNF	1-1.5 MDa

S4 Fig. SEC of HEK293T lysate suggests the salt-lability of mPDC in contrast to SWI-SNF subunits
(a) SEC profiles of mE1pα and mSWI-SNF subunits, BRG1 and BAF155 of HEK293T lysate in a buffer containing 350 mM NaCl. Elution volume in the SEC via Superose 6 column for every other 500 µl fraction is indicated with the expected size of eluted proteins based on standard proteins. (b) Comparison of the size of PDC subunits in 350 mM NaCl compared to other large complexes.

S5 Fig.

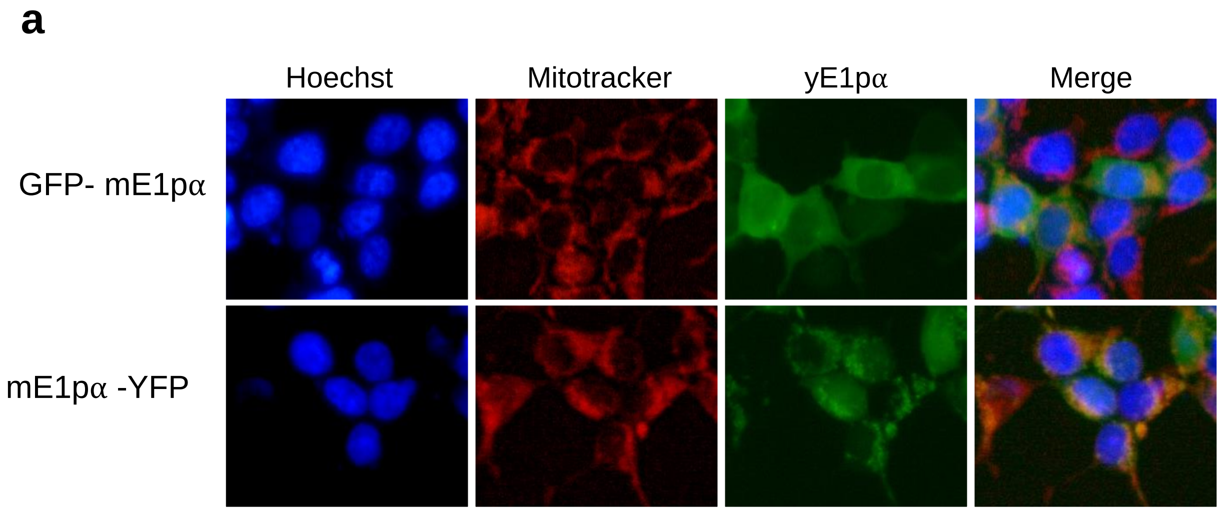

b

dNSAF	mE1pα-Halo
mE1pα (PDHA1)	0.1995
mE1pβ (PDHB1)	0.03162
mE2p (DLAT)	0.01221
mE3 (DLD)	0.001022
mE3BP (PDHX)	0.001654
PDHA2	0.001112

c

dNASF	Halo-mE1pα
mE1pα (PDHA1)	0.1531
mE1pβ (PDHB1)	0.004963
mE2p (DLAT)	0.000417
mE3 (DLD)	0
mE3BP (PDHX)	0
PDHA2	0.000164

S5 Fig. C-terminal but not N-terminally tagging allows correct localization of mPDC-E1α (PDHA1) and the purification of mPDC. (a) Microscopy images of HEK293T cells expressing GFP- E1α or E1α -YFP construct. For (b) and (c), relative protein levels (dNSAF) of mPDC components found in MudPIT analysis of (b) E1α-Halo and (c) Halo-E1α affinity purification.

S6 Fig.

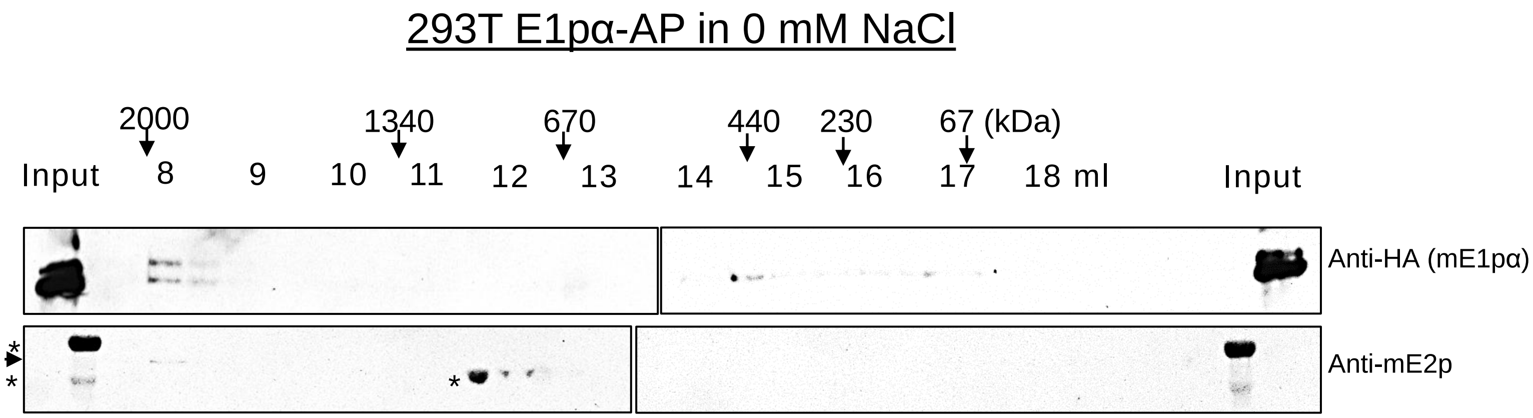

S6 Fig. SEC of Mammalian PDC in 0 mM NaCl display higher mw than that in 150 mM NaCl
Size fraction profiles of affinity purified mE1pα construct from HEK293T cells in a buffer containing 0 mM NaCl. Asterisks denote non-specific signals.

S7 Fig.

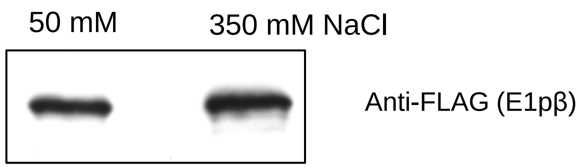

S7 Fig. Control experiment for catalytic activity assay. The amount of yPDC used in the catalytic activity assay for figure 4a measured by western blots against FLAG.

S1 Table. Yeast strains used in this study

Name	Genotype	Source
Wildtype (By4741)	MATa his3Δ1 leu2Δ0 met15Δ0 ura3Δ0	Open Biosystems
E1pβ-5xFLAG (Pdb1-5xFLAG)	MATa his3Δ1 leu2Δ0 met15Δ0 ura3Δ0 Pdb1-5xFLAG::NatMX4	This study
E3-5xFLAG (Lpd1-5xFLAG)	MATa his3Δ1 leu2Δ0 met15Δ0 ura3Δ0 Lpd1-5xFLAG::NatMX4	This study
E1pβ-3xHA/ E2p-V5 (Pdb1-3xHA/Lat1-V5)	MATa his3Δ1 leu2Δ0 met15Δ0 ura3Δ0 Pdb1-3xHA::KanMX4 Lat1-V5::His3	This study
E2p-V5/ E3-5xFLAG (Lat1-V5/Lpd1-5xFLAG)	MATa his3Δ1 leu2Δ0 met15Δ0 ura3Δ0 Lat1-V5::His3 LPD1-5xFLAG::NatMX4	This study
E1pβ-3xHA/ E2p-V5/ E3-5xFLAG (Pdb1-3xHA/Lat1-V5/ Lpd1-5xFLAG)	MATa his3Δ1 leu2Δ0 met15Δ0 ura3Δ0 Pdb1-3xHA::KanMX4 Lat1-V5::His3 Lpd1-5xFLAG::NatMX4	This study
*pkp2*Δ/PDB1-5xFLAG	MATa his3Δ1 leu2Δ0 met15Δ0 ura3Δ0 pkp2Δ::KanMX4 Pdb1-5XFLAG::NatMX4	This study
E1pβΔ (*pdb1*Δ)	MATa his3Δ1 leu2Δ0 met15Δ0 ura3Δ0 pdb1Δ::KanMX4	Open Biosystems
E2pΔ (*lat1*Δ)	MATa his3Δ1 leu2Δ0 met15Δ0 ura3Δ0 lat1Δ::KanMX4	Open Biosystems
E3Δ (*lat1*Δ)	MATa his3Δ1 leu2Δ0 met15Δ0 ura3Δ0 lpd1Δ::KanMX4	Open Biosystems
