# Supplementary material for: The plasticity of the pyruvate dehydrogenase complex confers a labile structure that is associated with its catalytic activity
Source: PLoS One. 2020 Dec 28;15(12):e0243489. doi: 10.1371/journal.pone.0243489 (PMC7769281; doi:10.1371/journal.pone.0243489)

For Fig.1b, Anti-FLAG blots are shown below.
Size fractions of Pdb1-3xHA/Lat1-V5/Lpd1-5xFLAG from HS extract in a buffer containing 0 mM NaCl with the elution volumes in the SEC via Superose 6 column for every 500 μl fraction indicated.

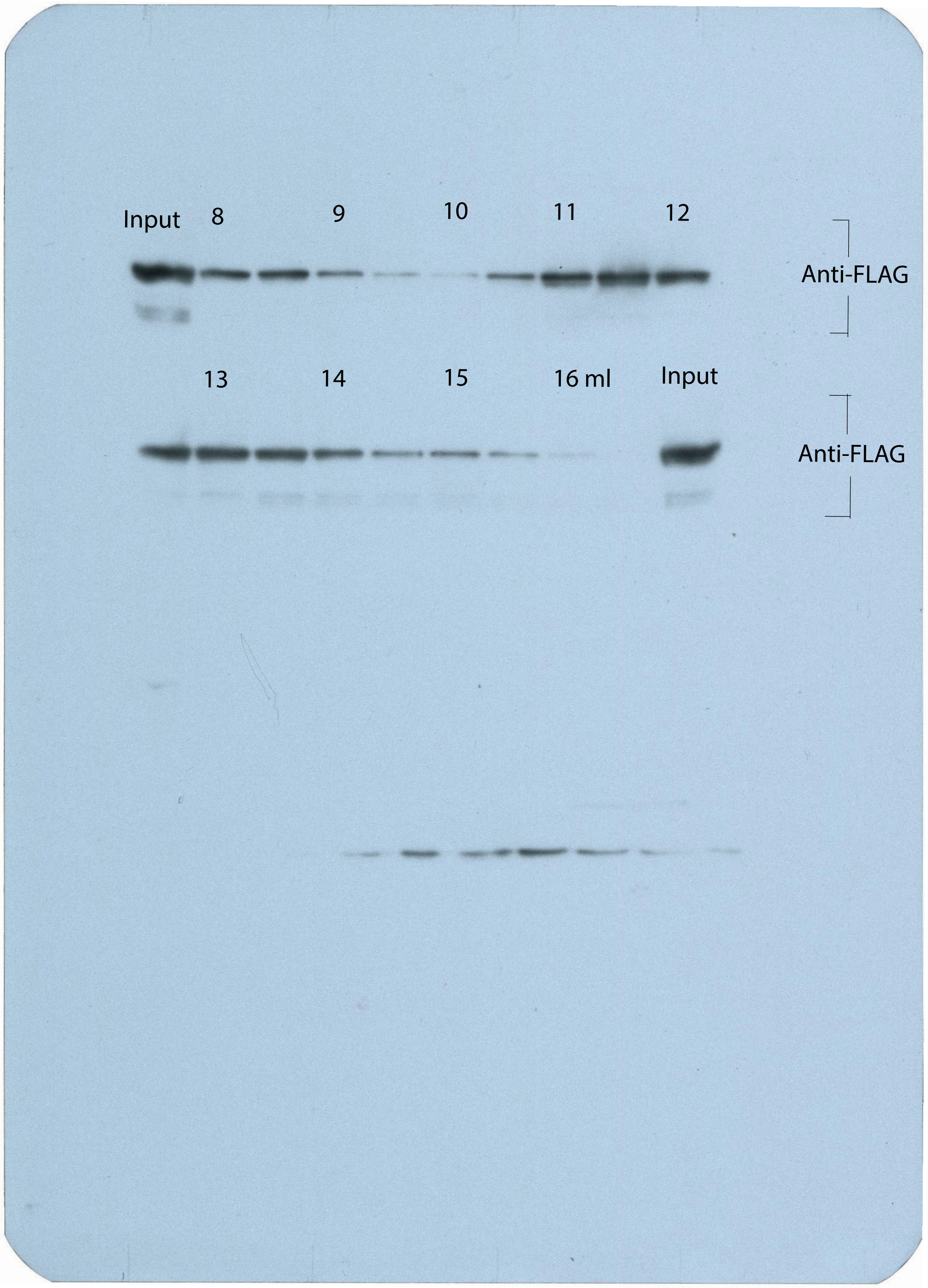

For Fig.1b, Anti-V5 and Anti-HA blots are shown.
Size fractions of Pdb1-3xHA/Lat1-V5/Lpd1-5xFLAG from HS extract in a buffer containing 0 mM NaCl with the elution volumes in the SEC via Superose 6 column for every 500 µl fraction indicated.

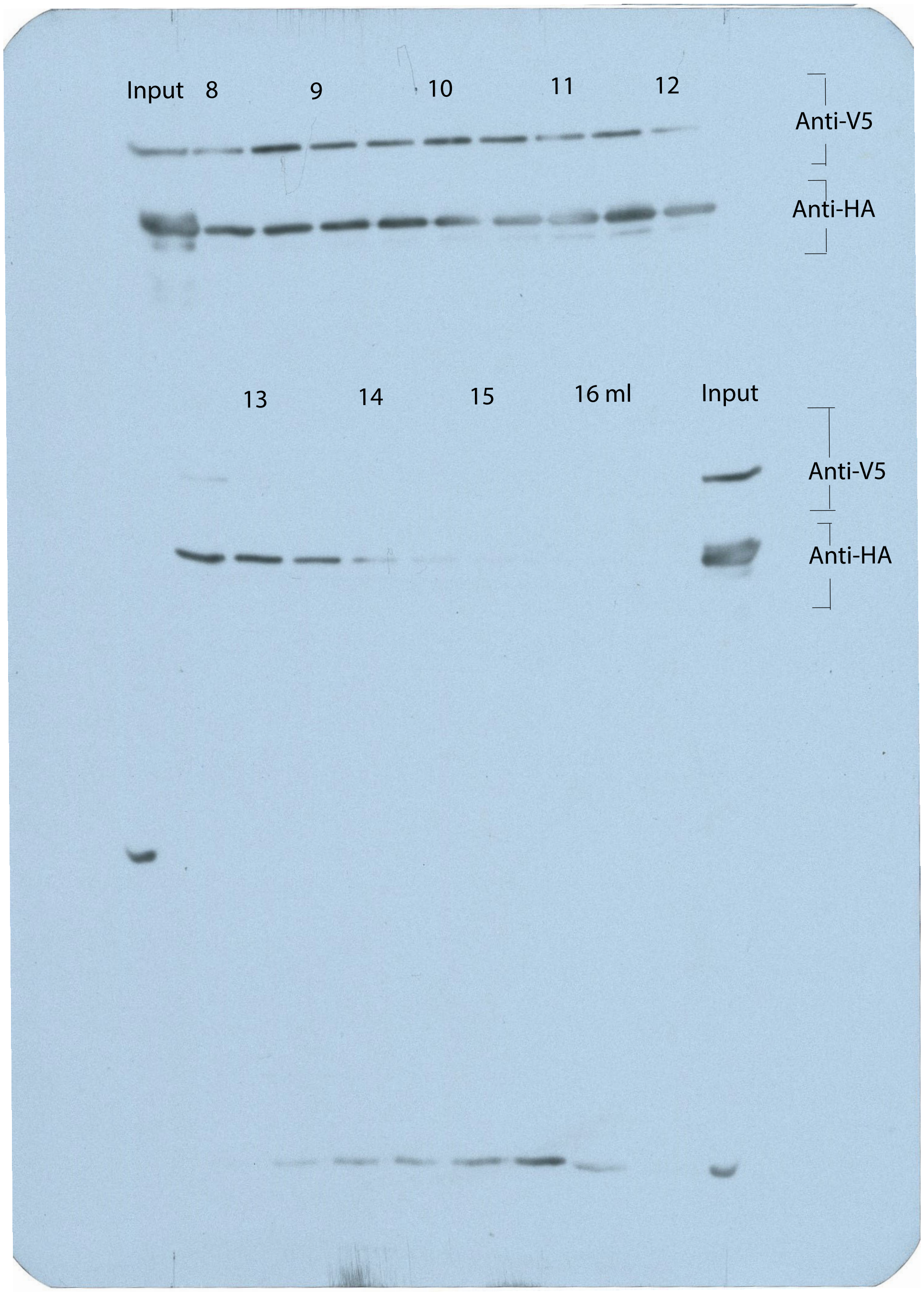

For Fig.1c, Anti-FLAG and Anti-HA blots are shown below.
Size fractions of Pdb1-3xHA/Lat1-V5/Lpd1-5xFLAG from LS extract in a buffer containing 0 mM NaCl with the elution volumes in the SEC via Superose 6 column for every 500 µl fraction indicated.

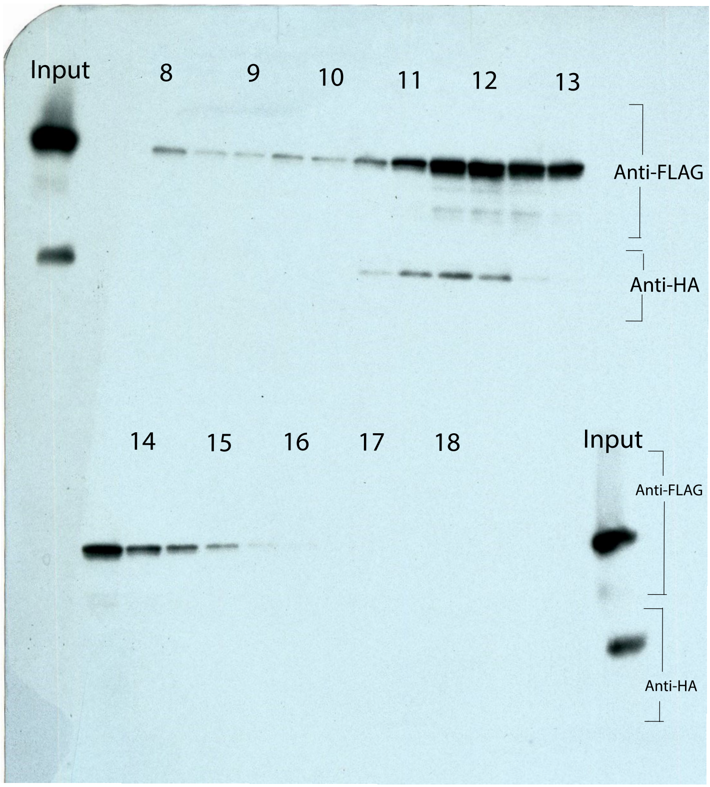

For Fig.1c, Anti-V5 blots are shown below.
Size fractions of Pdb1-3xHA/Lat1-V5/Lpd1-5xFLAG from LS extract in a buffer containing 0 mM NaCl with the elution volumes in the SEC via Superose 6 column for every 500 µl fraction indicated.

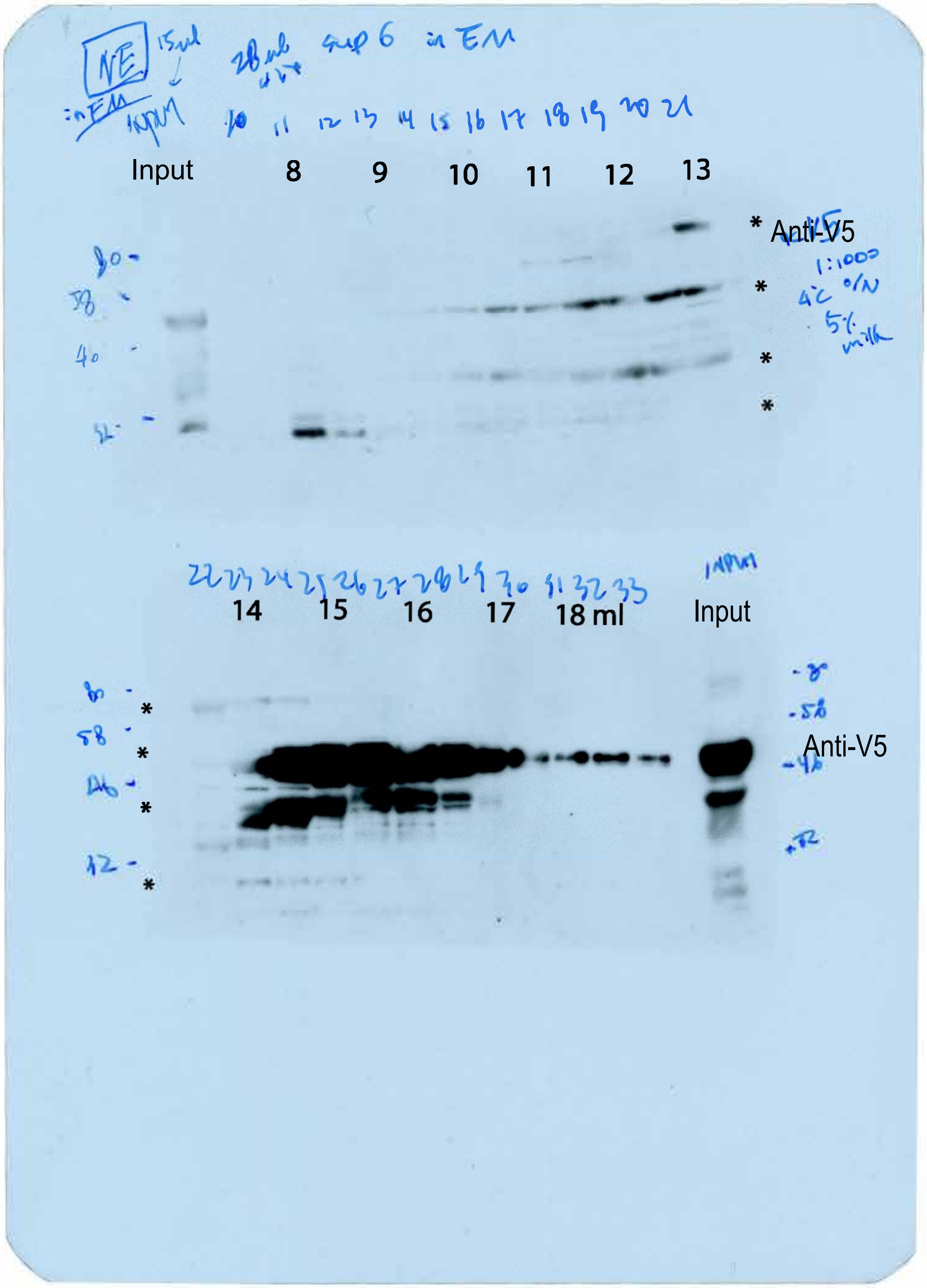

For Fig.1d, Anti-FLAG and Anti-HA blots are shown below.
Size fractions of Pdb1-3xHA/Lat1-V5/Lpd1-5xFLAG from HS extract in a buffer containing 350 mM NaCl with the elution volumes in the SEC via Superose 6 column for every 500 μl fraction indicated.

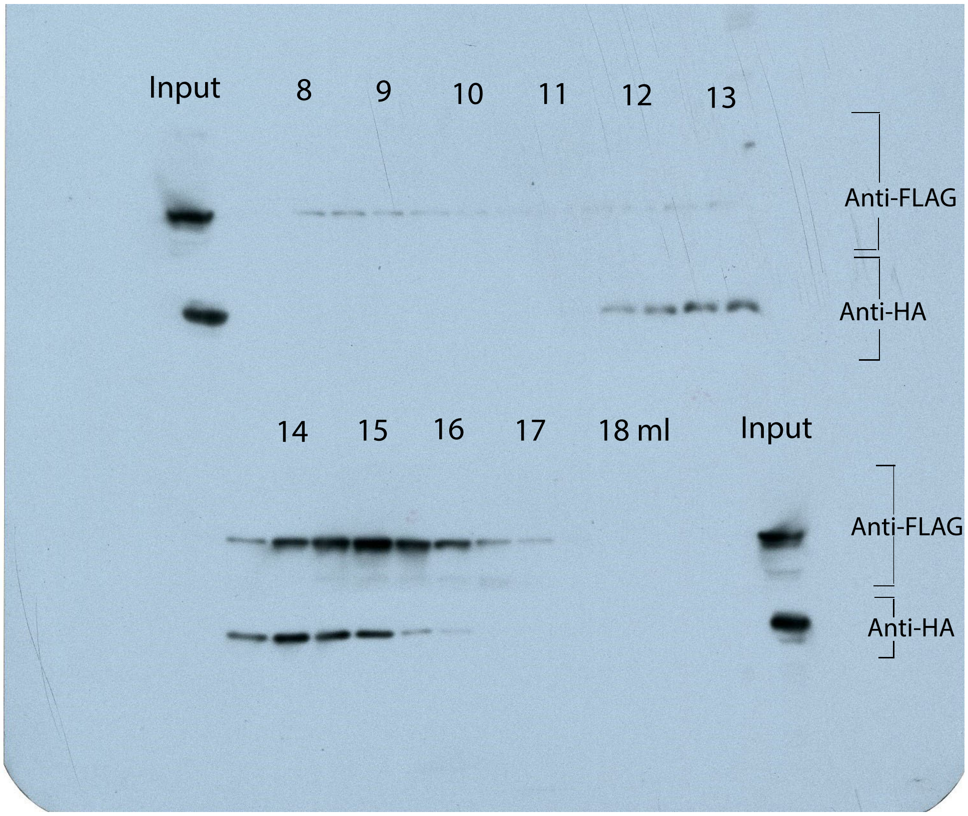

For Fig.1d, Anti-V5 blots are shown below.
Size fractions of Pdb1-3xHA/Lat1-V5/Lpd1-5xFLAG from HS extract in a buffer containing 350 mM NaCl with the elution volumes in the SEC via Superose 6 column for every 500 μl fraction indicated.

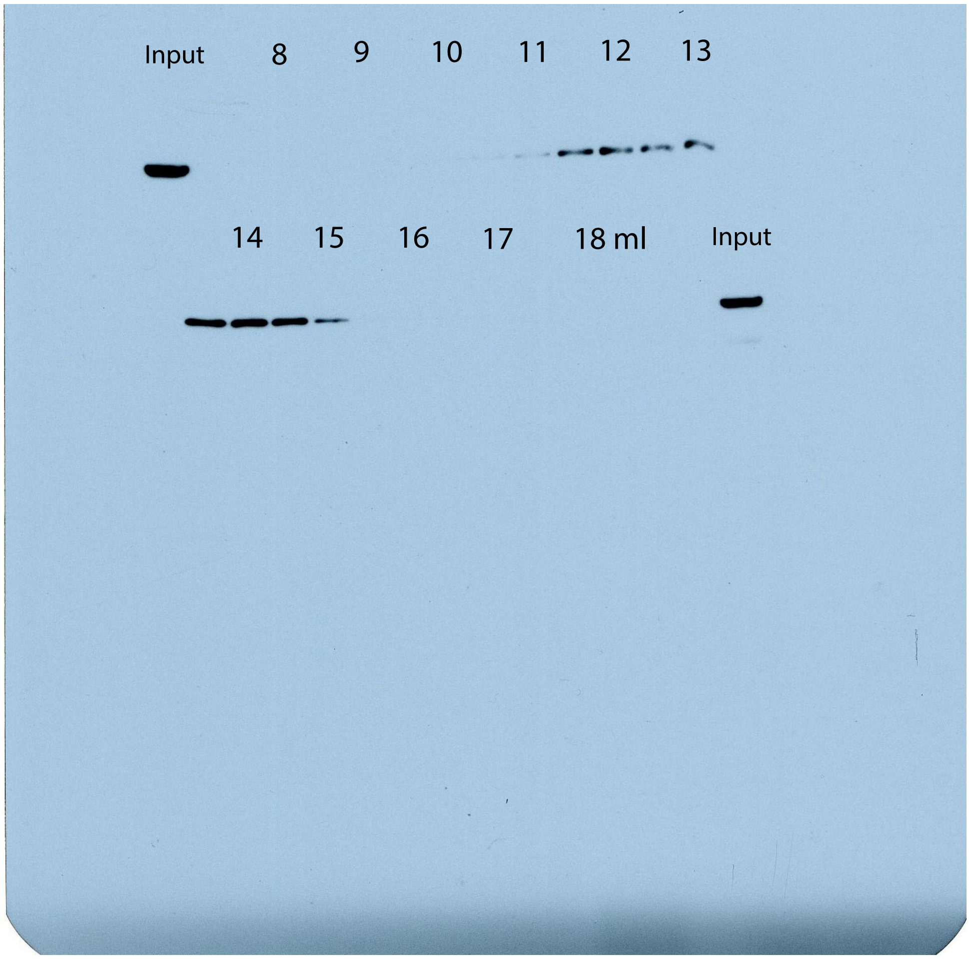

For Fig.1e, Anti-V5 blots are shown below.
Size fractions of Pdb1-3xHA/Lat1-V5/Lpd1-5xFLAG from LS extract
in a buffer containing 350 mM NaCl with the elution volumes in the SEC via
Superose 6 column for every 500 µl fraction indicated.

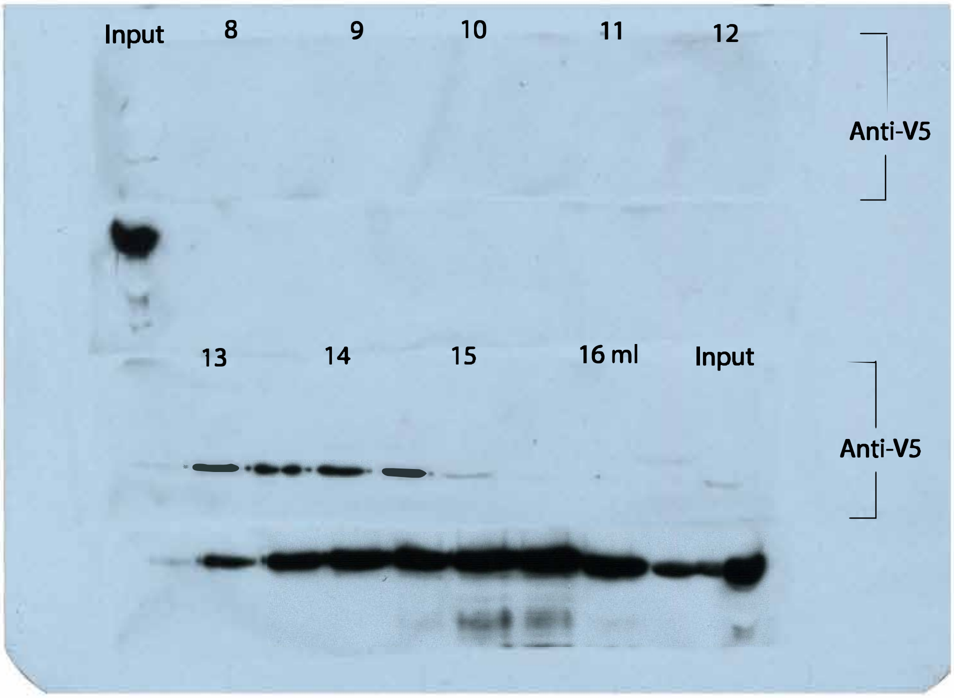

For Fig.1e, Anti-FLAG blots are shown below.
Size fractions of Pdb1-3xHA/Lat1-V5/Lpd1-5xFLAG from LS extract in a buffer containing 350 mM NaCl with the elution volumes in the SEC via Superose 6 column for every 500 µl fraction indicated.

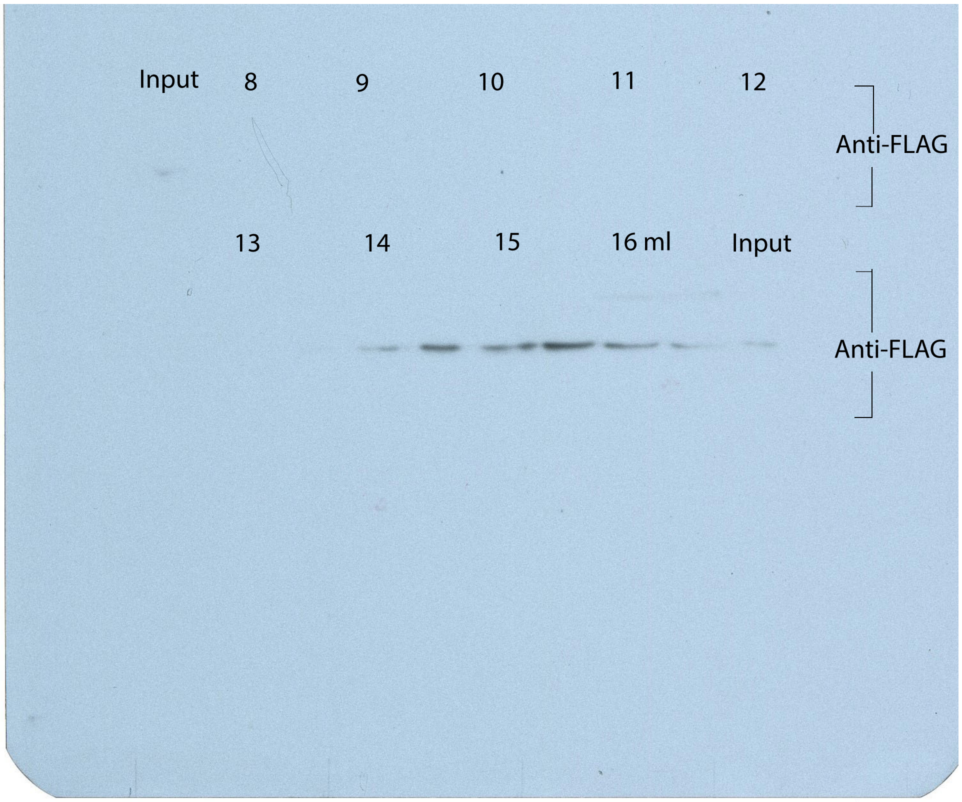

For Fig.1e, Anti-HA blots are shown below.
Size fractions of Pdb1-3xHA/Lat1-V5/Lpd1-5xFLAG from LS extract in a buffer containing 350 mM NaCl with the elution volumes in the SEC via Superose 6 column for every 500 µl fraction indicated.

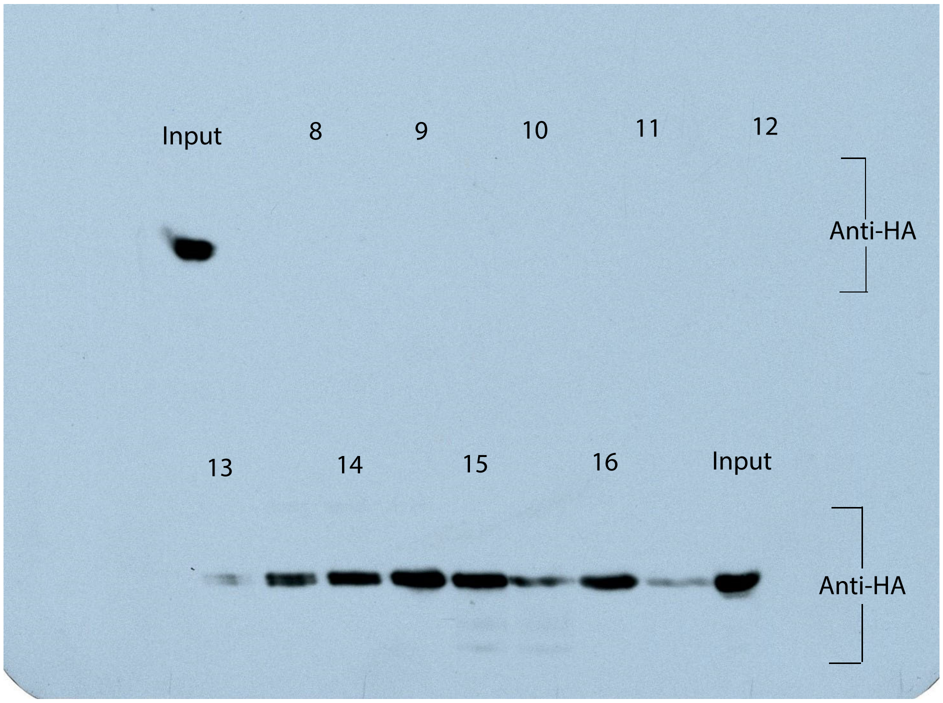

For Fig.2a, Anti-FLAG blots are shown below.
Size fractions of FLAG purified Pdb1-5xFLAG in a buffer containing
350 mM NaCl with the elution volumes in the SEC via Superose 6 column
for every 500 µl fraction indicated.

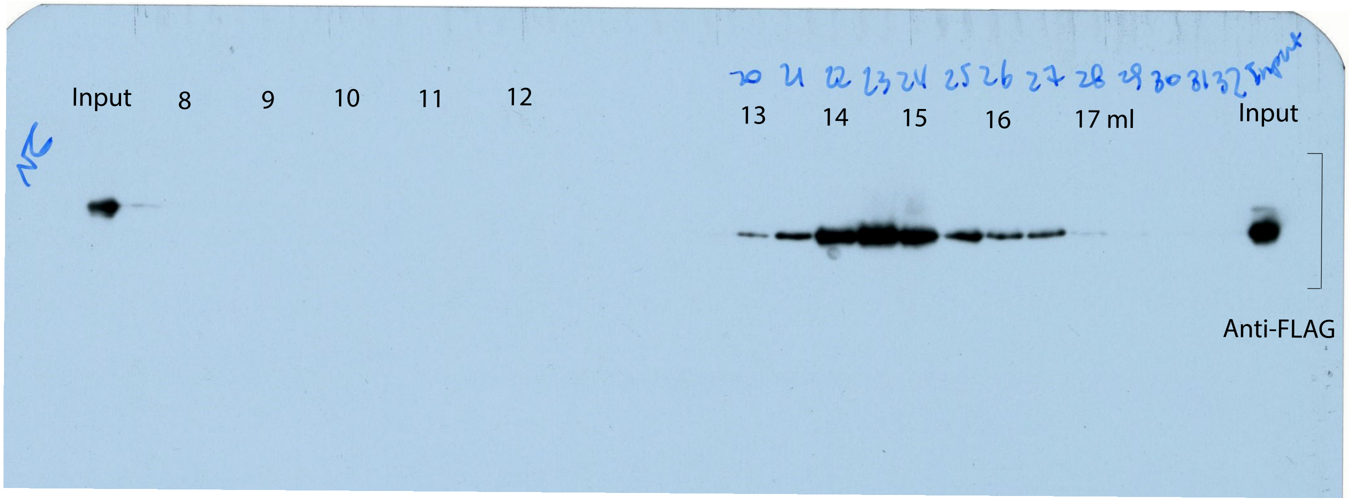

For Fig.2b, Anti-FLAG blots are shown below.
Size fractions of FLAG purified Lpd1-5xFLAG in a buffer containing 350 mM NaCl with the elution volumes in the SEC via Superose 6 column for every 500 μl fraction indicated.

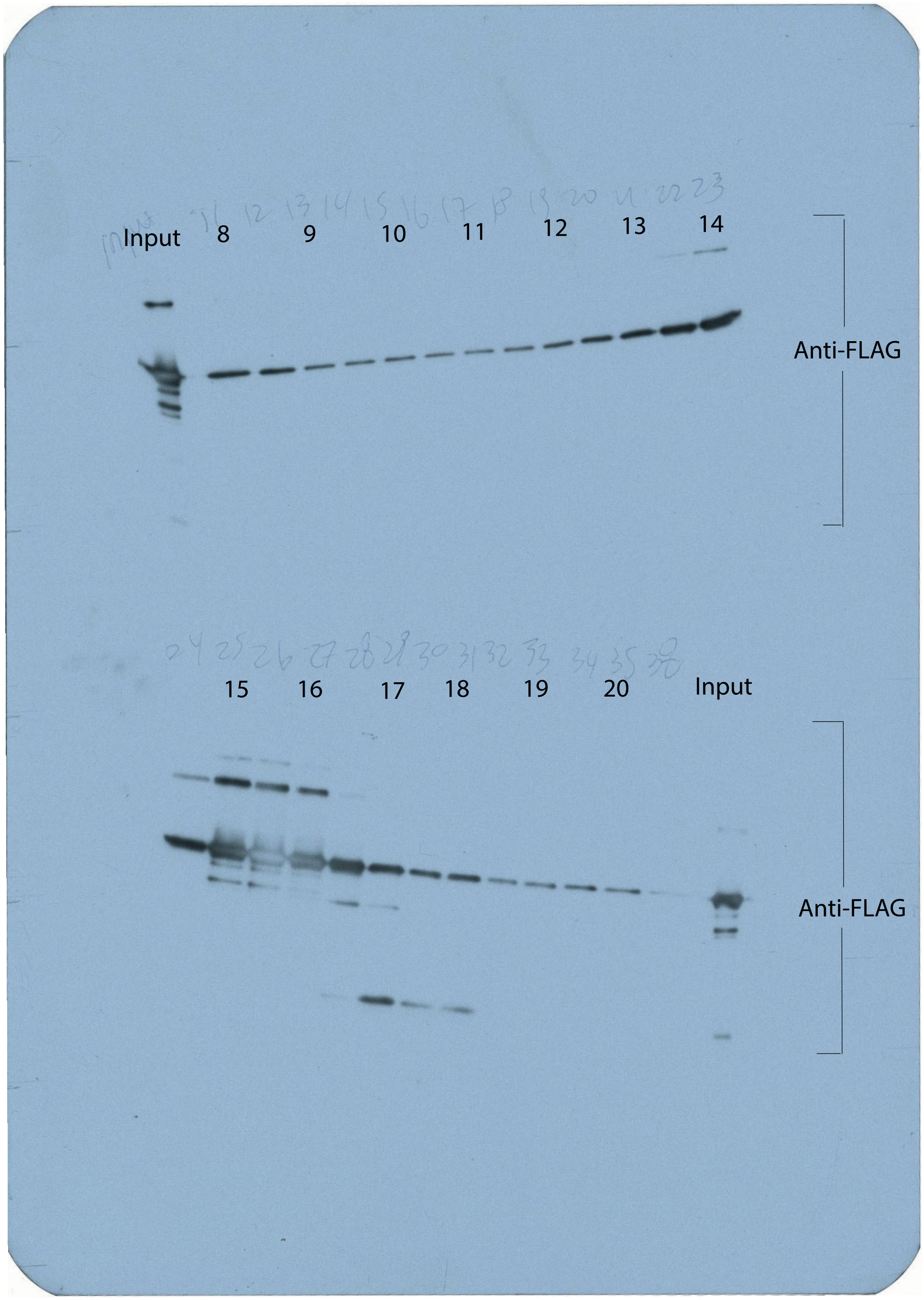

For Fig.2c, Anti-FLAG blots are shown below.
IP of E1β-3xHA in Lat1-V5/Lpd1-5xFLAG and Pdb1-3xHA/Lat1-V5/Lpd1-5xFLAG in the presence of 0 mM, 150 mM, or 350 mM NaCl. Input samples (whole cell extract) and IP samples were loaded.

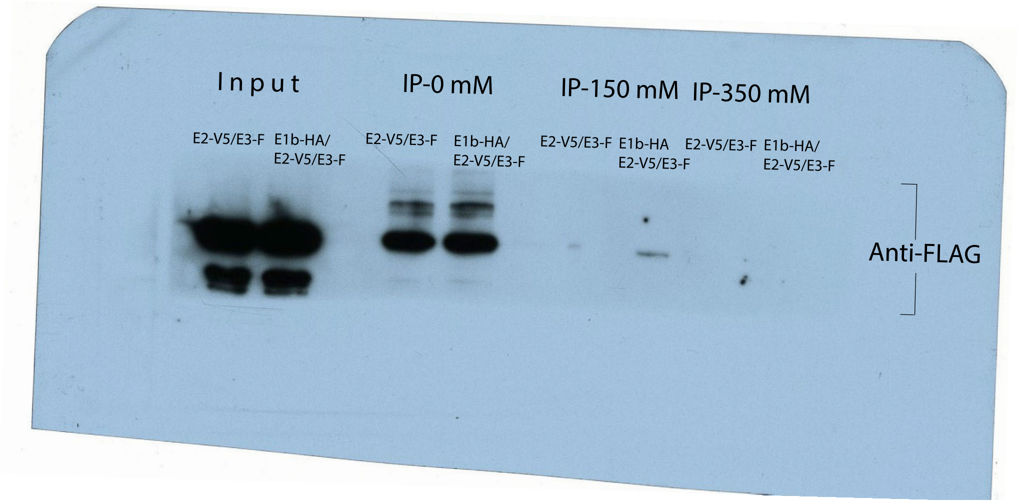

For Fig.2c, Anti-V5 and Anti-HA blots are shown below.
IP of E1β-3xHA in E2-V5/E3-5xFLAG and Pdb1-3xHA/Lat1-V5/Lpd1-5xFLAG in the presence of 0 mM, 150 mM, or 350 mM NaCl. Input samples (whole cell extract) and IP samples were loaded.

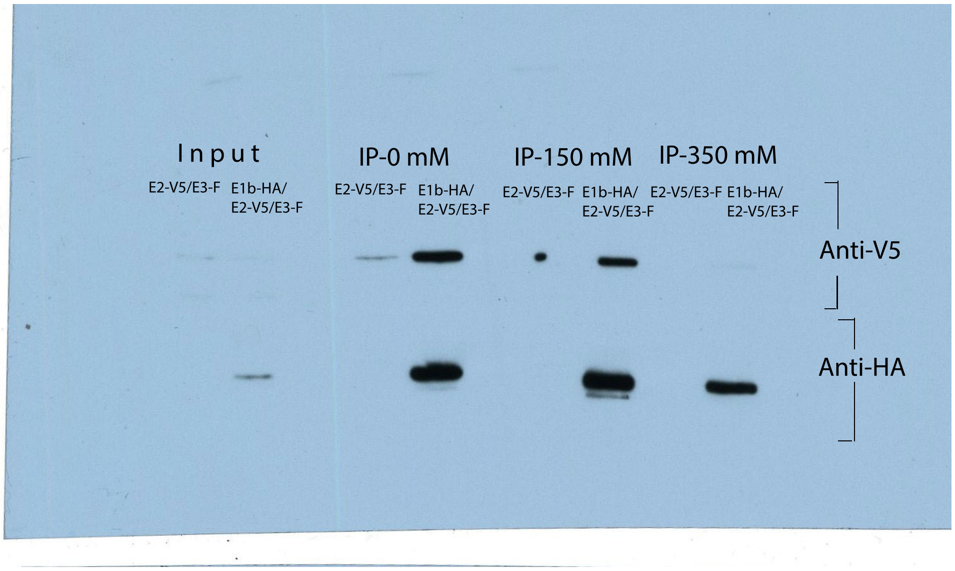

For Fig.2d, Anti-FLAG blots are shown below.
Size fractions of FLAG purified Pdb1-3xHA/Lat1-V5/Lpd1-5xFLAG in a buffer containing 0 mM NaCl with the elution volumes in the SEC via Superose 6 column for every 500 µl fraction indicated.

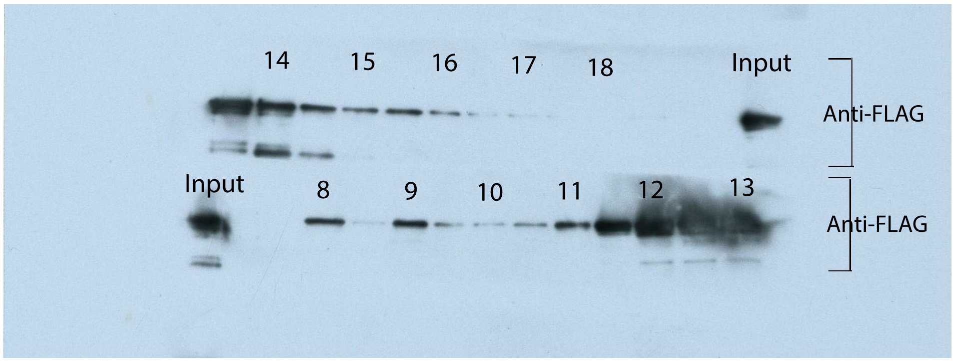

For Fig.2d, Anti-V5 blots are shown below.
Size fractions of FLAG purified Pdb1-3xHA/Lat1-V5/Lpd1-5xFLAG in a buffer containing 0 mM NaCl with the elution volumes in the SEC via Superose 6 column for every 500 μl fraction indicated.

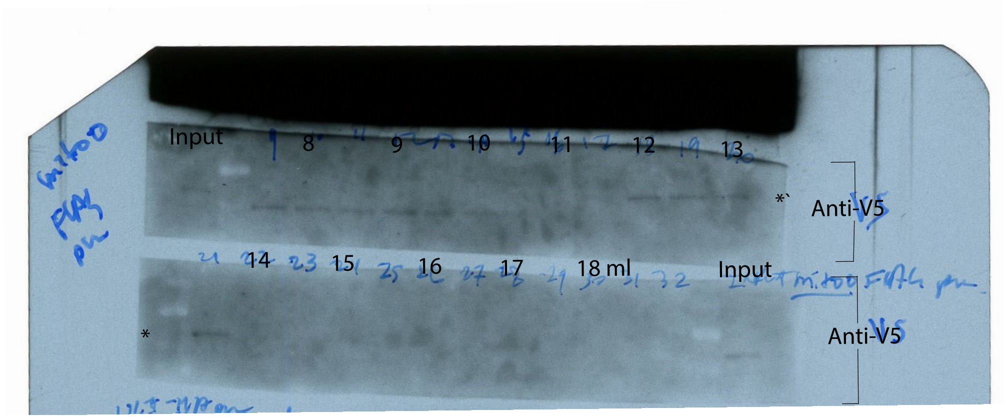

For Fig.2d, Anti-HA blots are shown below.
Size fractions of FLAG purified Pdb1-3xHA/Lat1-V5/Lpd1-5xFLAG
in a buffer containing 0 mM NaCl with the elution volumes in the SEC via
Superose 6 column for every 500 µl fraction indicated.

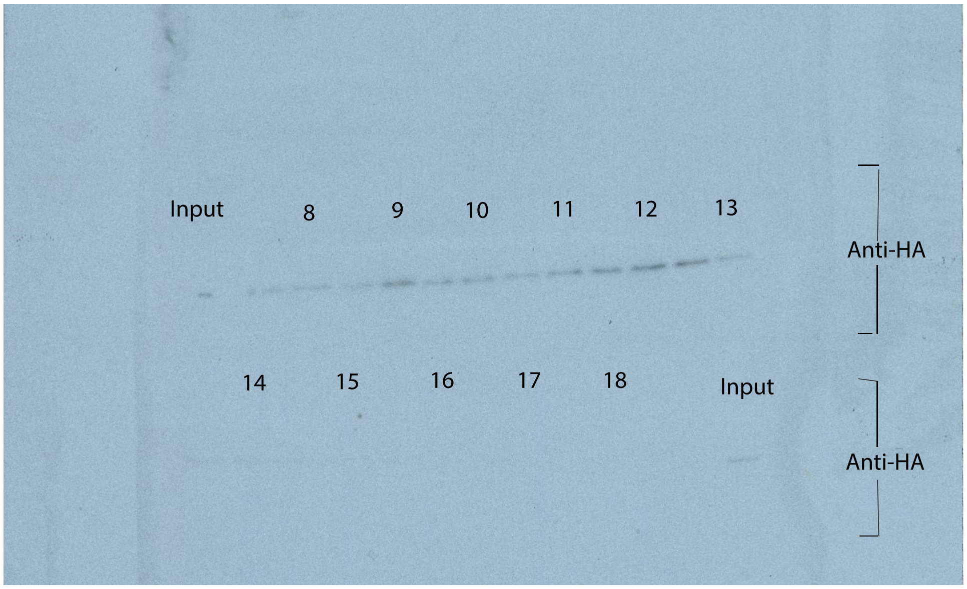

For Fig.3a, Anti-mE1pa blots are shown below.
Size fractions of HEK293T lysates in a buffer containing 350 mM NaCl with the elution volumes in the SEC via Superose 6 column for every 500 µl fraction indicated.

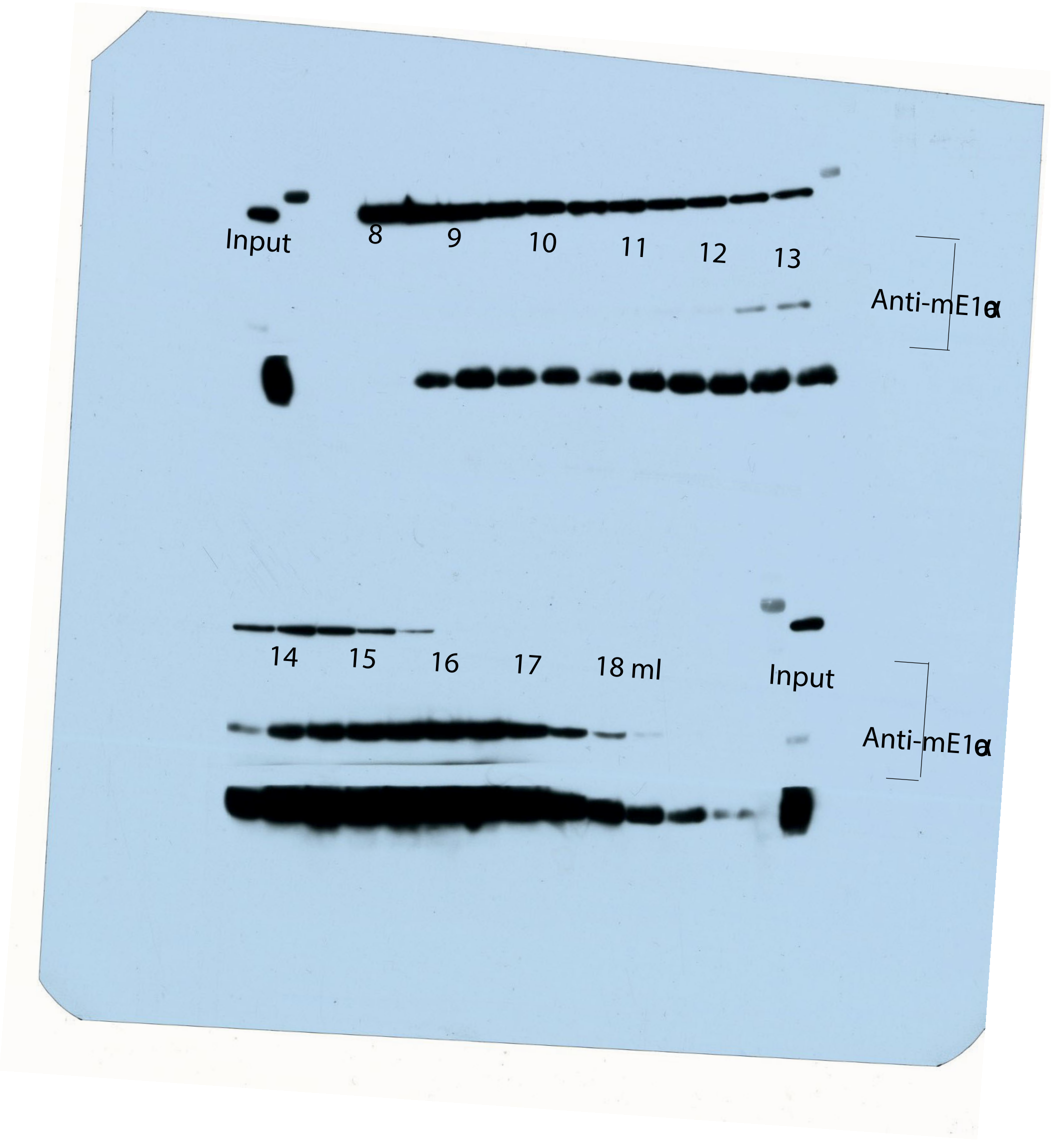

For Fig.3a, Anti-mE2p and Anti-mE1pb blots are shown below.
Size fractions of HEK293T lysates in a buffer containing 350 mM NaCl with the elution volumes in the SEC via Superose 6 column for every 500 μl fraction indicated.

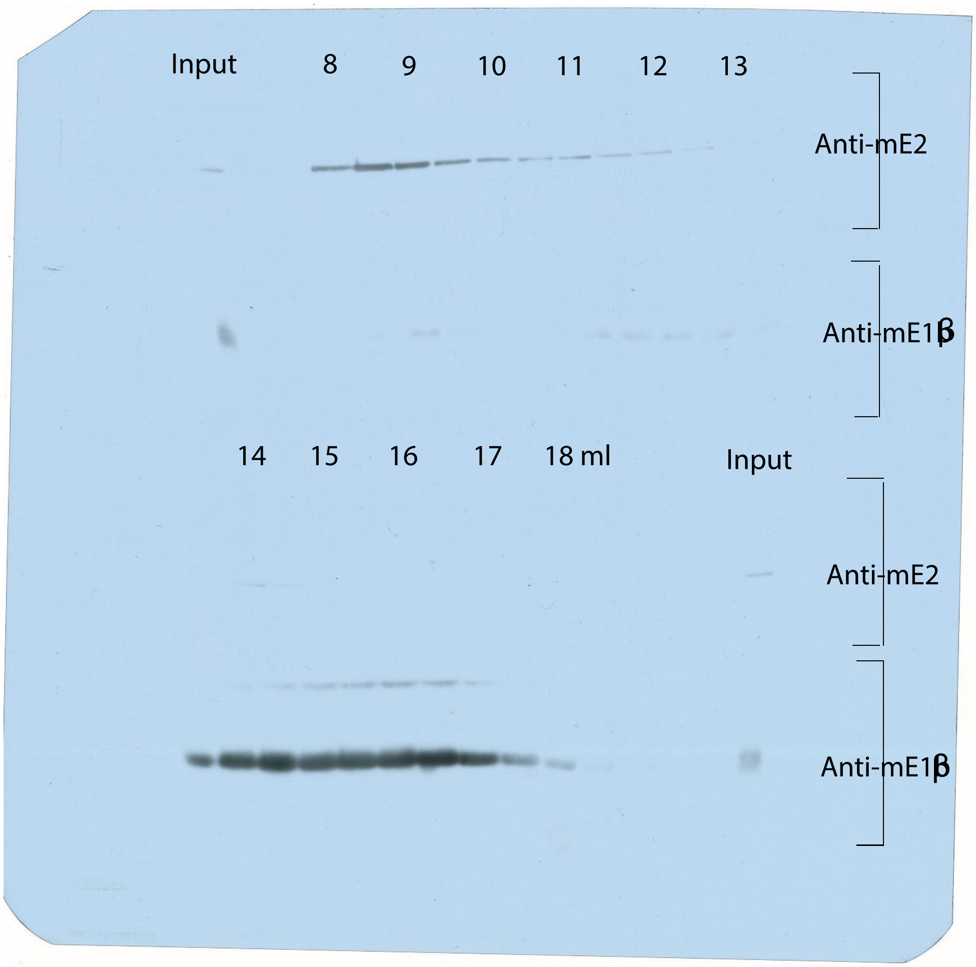

For Fig.3a, Anti-mE3 blots are shown below.
Size fractions of HEK293T lysates in a buffer containing 350 mM NaCl with the elution volumes in the SEC via Superose 6 column for every 500 μl fraction indicated.

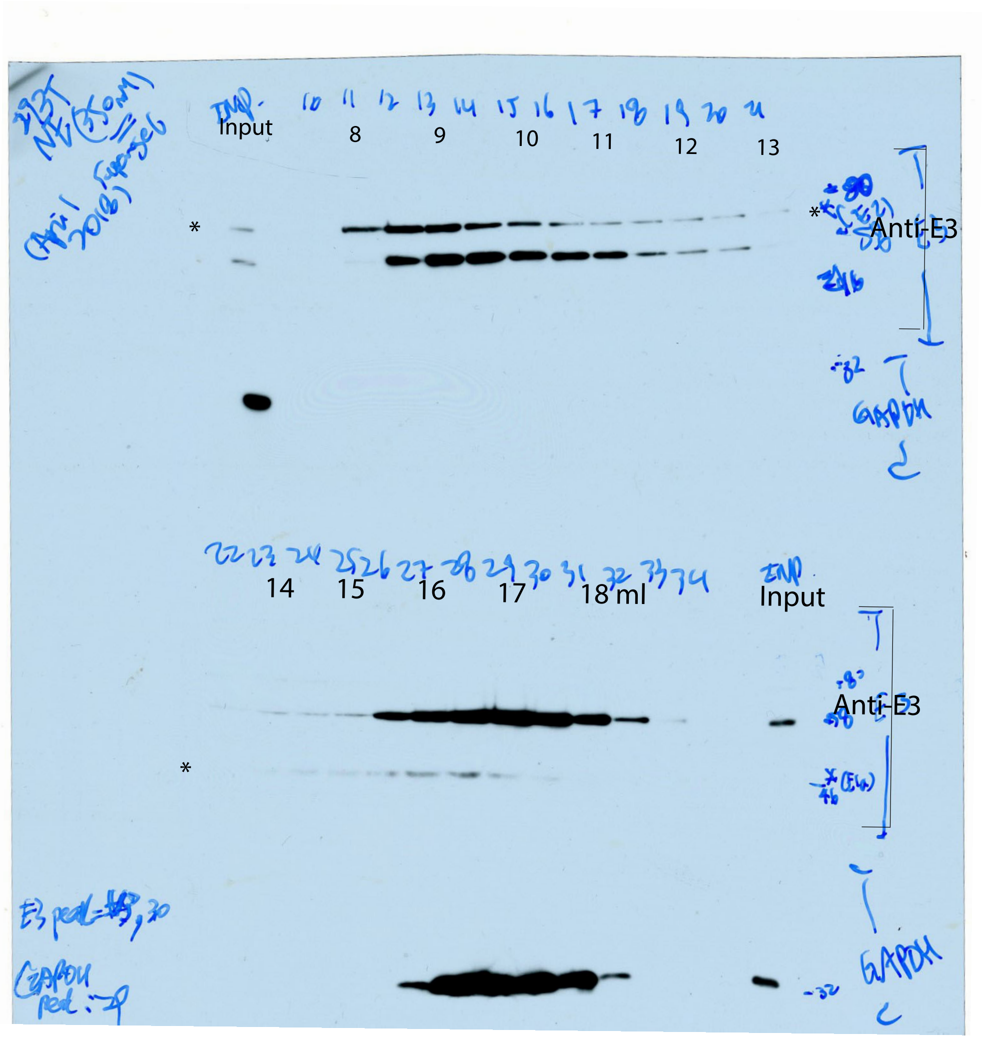

For Fig.3b, Anti-HA blots are shown below.
Size fractions of Halo-purified PDHA1-HA ectopically expressing in HEK293T lysates in a buffer containing 350 mM NaCl with the elution volumes in the SEC via Superose 6 column for every 500 µl fraction indicated.

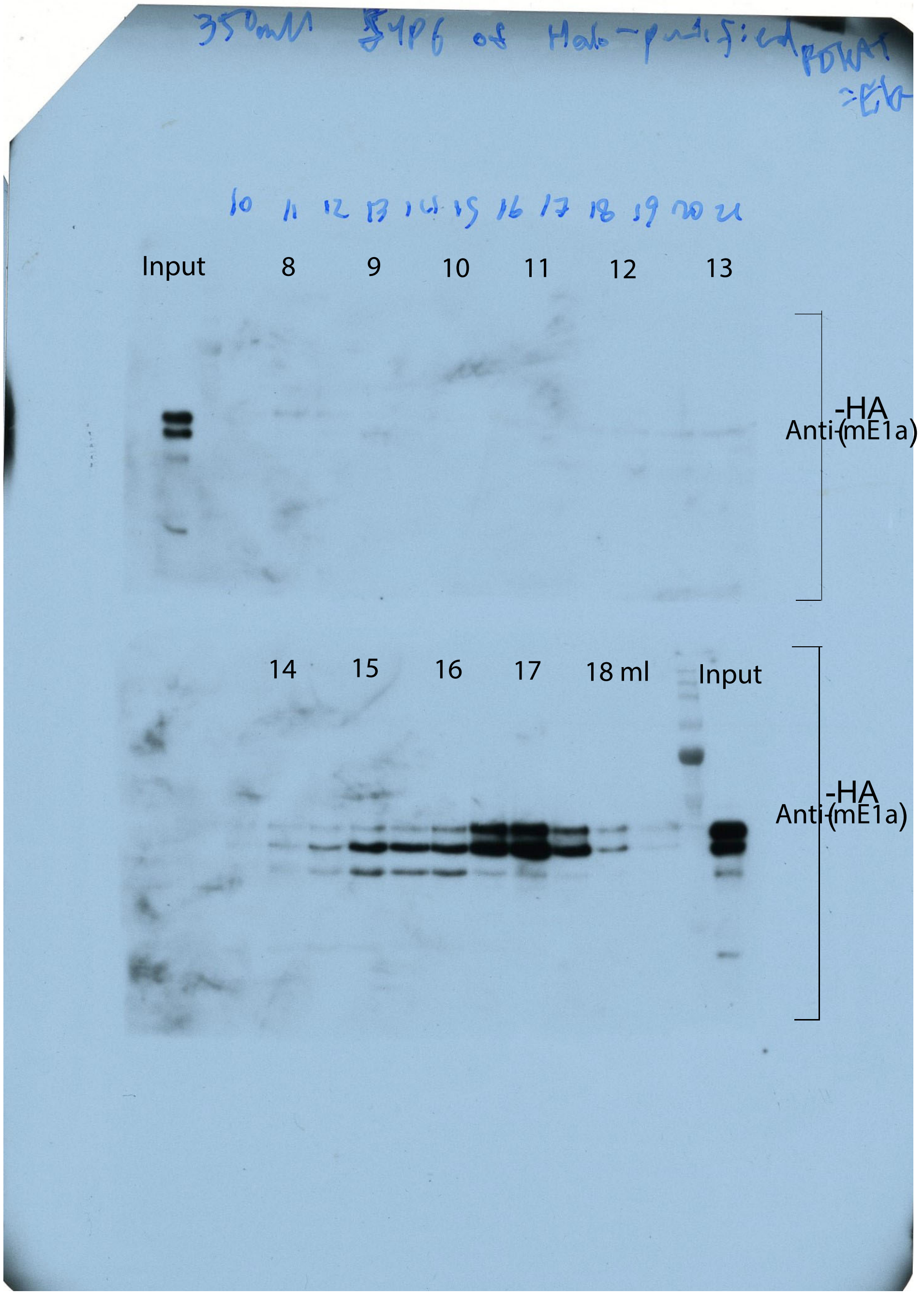

For Fig.3b, Anti-mE2p and Anti-mE1pβ blots are shown below.
Size fractions of Halo-purified PDHA1-HA ectopically expressing in HEK293T lysates in a buffer containing 350 mM NaCl with the elution volumes in the SEC via Superose 6 column for every 500 μl fraction indicated

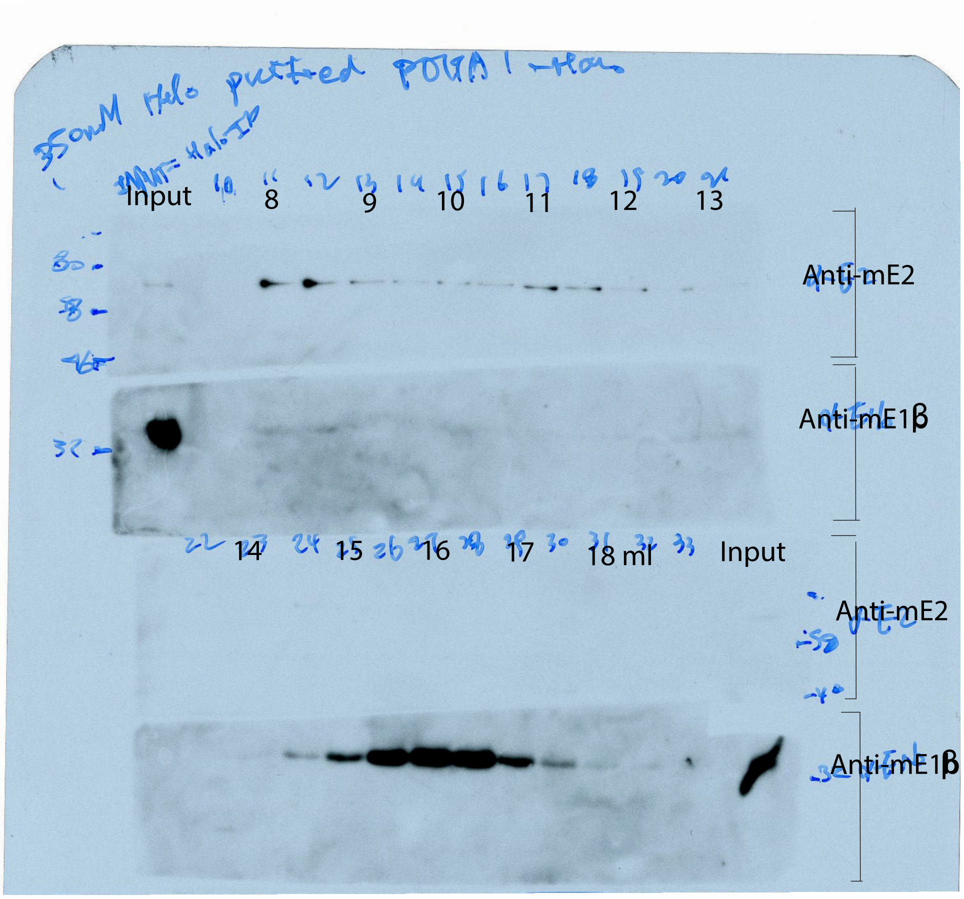

For Fig.3c, Anti-HA blots are shown below.
Size fractions of Halo-purified mE1pα (PDHA1-HA) ectopically expressing in HEK293T lysates in a buffer containing 150 mM NaCl with the elution volumes in the SEC via Superose 6 column for every 500 μl fraction indicated

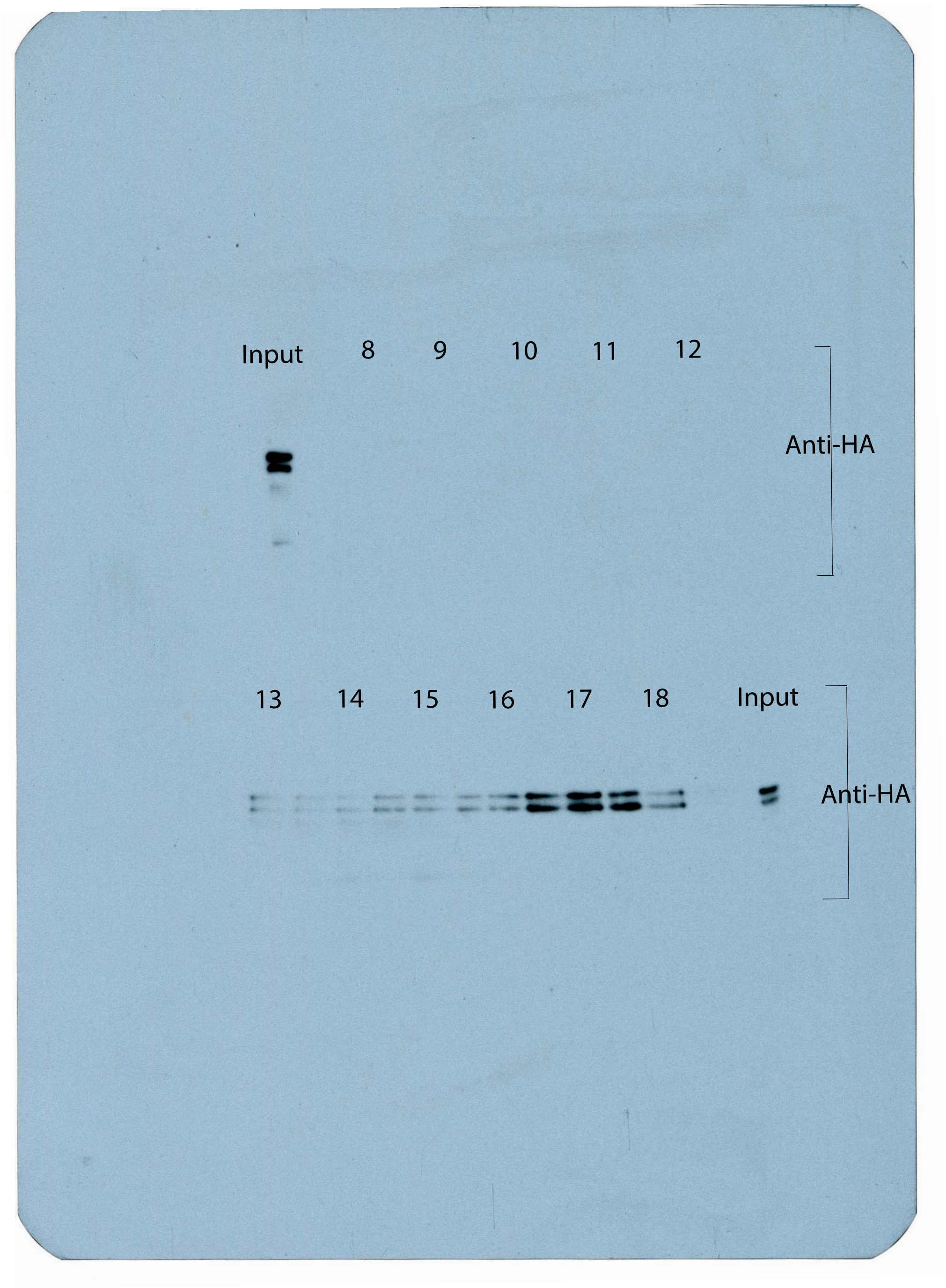

For Fig.3c, Anti-mE1pβ blots are shown below.
Size fractions of Halo-purified mE1pα (PDHA1-HA) ectopically expressing in HEK293T lysates in a buffer containing 150 mM NaCl with the elution volumes in the SEC via Superose 6 column for every 500 μl fraction indicated

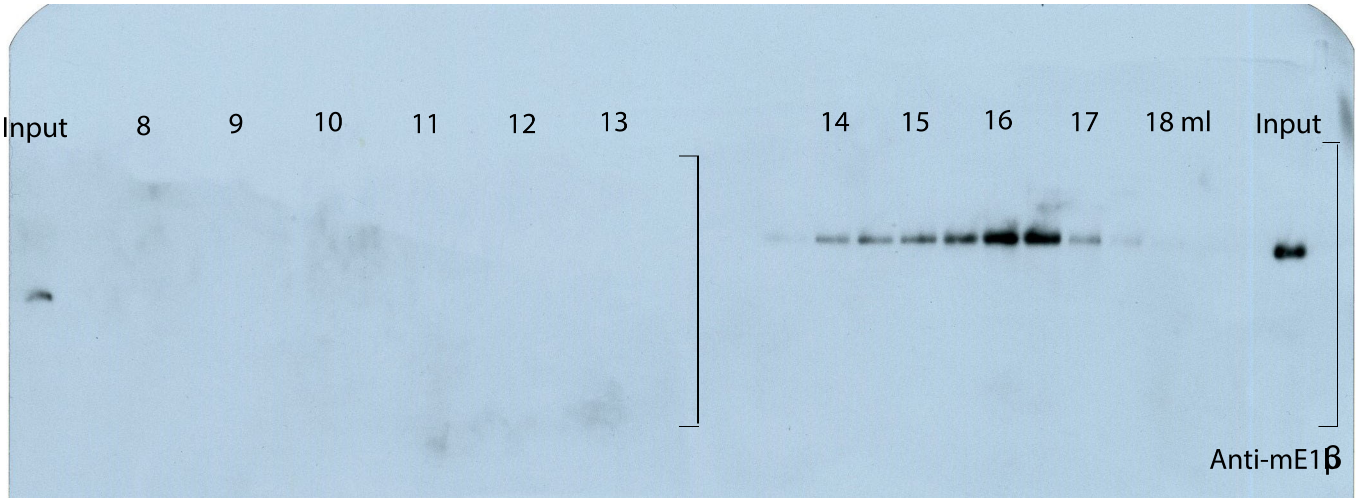

For Fig.3c, Anti-mE2p blots are shown below.
Size fractions of Halo-purified mE1pα (PDHA1-HA) ectopically expressing in HEK293T lysates in a buffer containing 150 mM NaCl with the elution volumes in the SEC via Superose 6 column for every 500 µl fraction indicated

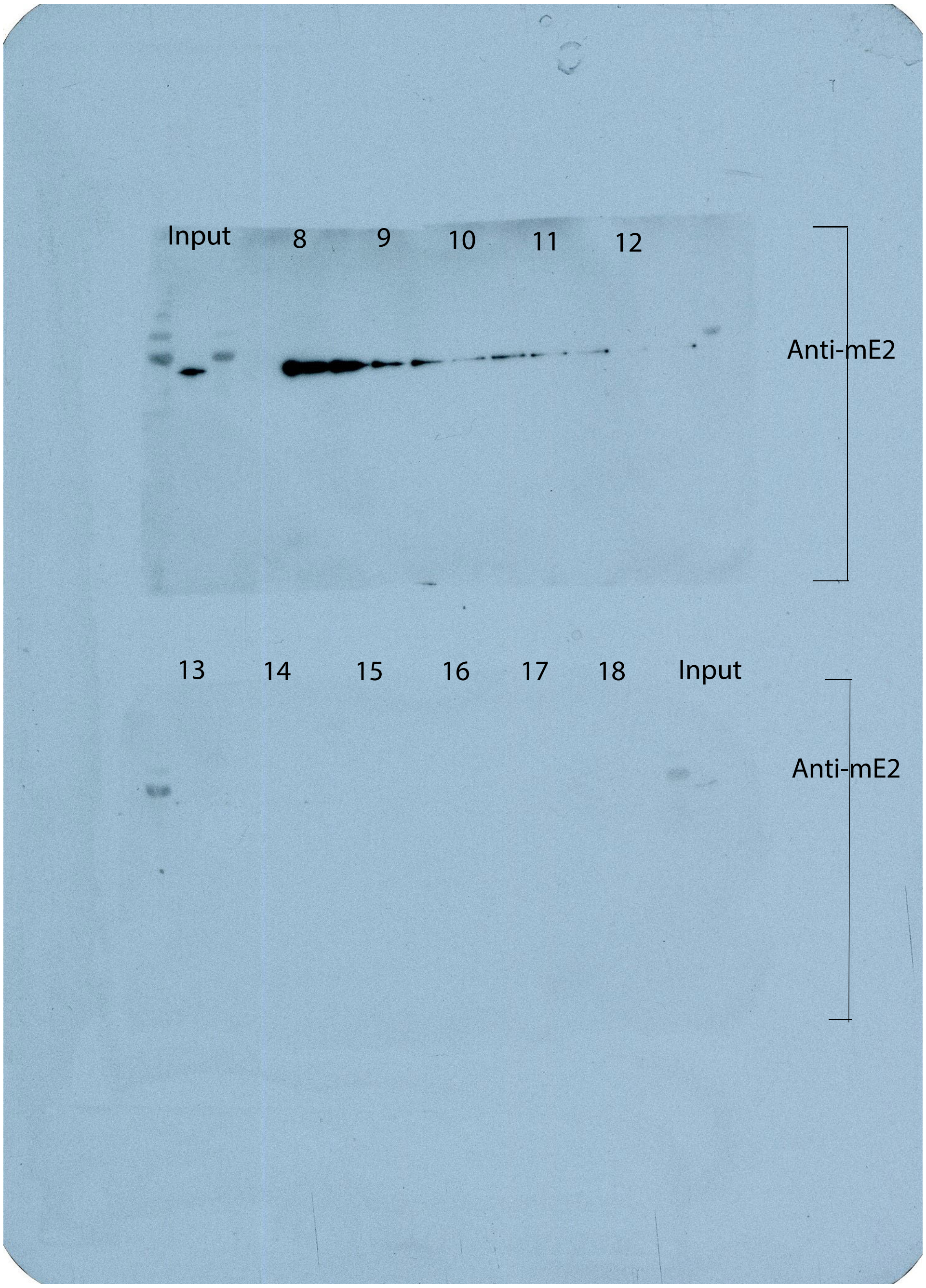

For Fig.S2b, Anti-FLAG blots are shown below.
Size fractions of Pdb1-3xHA/Lat1-V5/Lpd1-5xFLAG from whole cell extract in a buffer containing 50 mM NaCl with the elution volumes in the SEC via Superose 6 column for every other 500 µl fraction indicated.

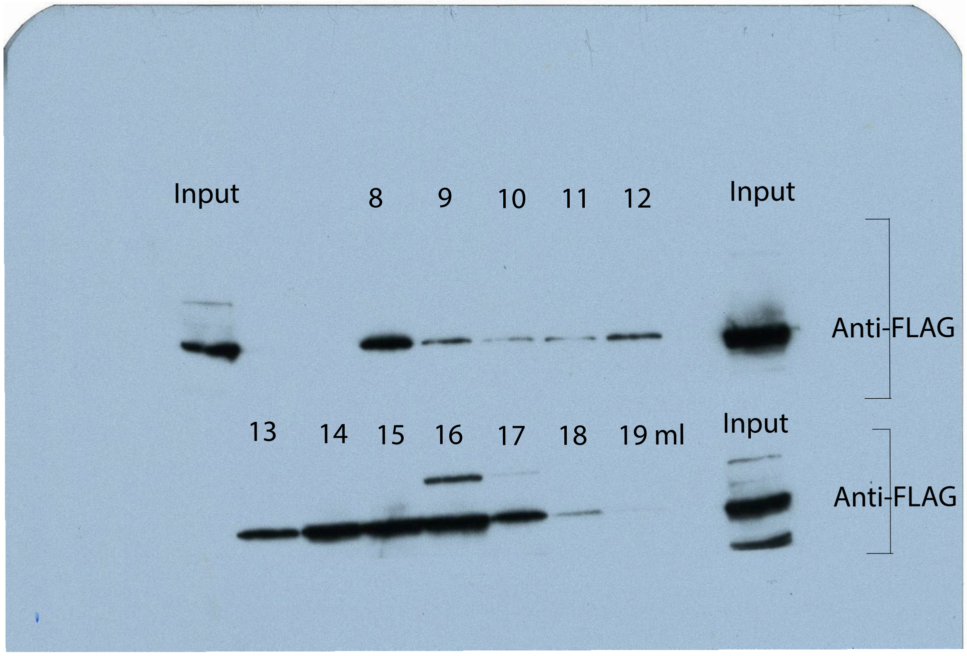

For Fig.S2b, Anti-HA blots are shown below.
Size fractions of Pdb1-3xHA/Lat1-V5/Lpd1-5xFLAG from whole cell extract in a buffer containing 50 mM NaCl with the elution volumes in the SEC via Superose 6 column for every other 500 µl fraction indicated.

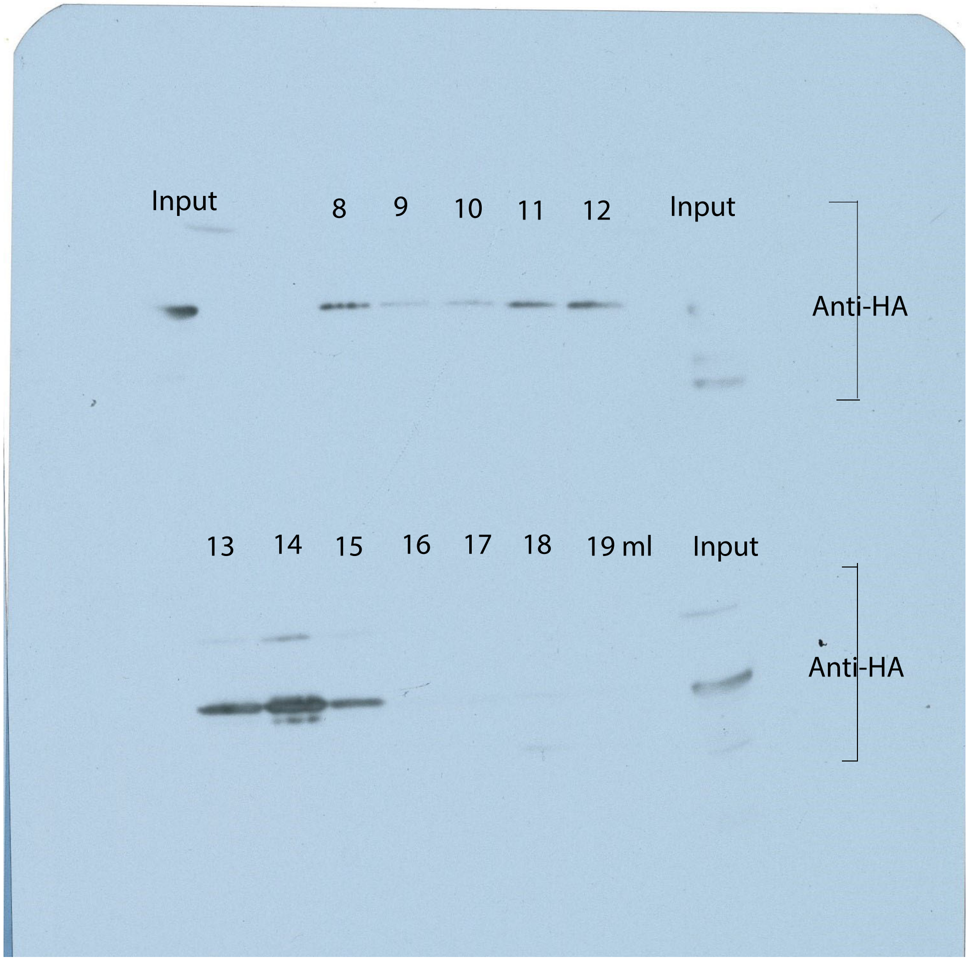

For Fig.S2b, Anti-V5 blots are shown below.
Size fractions of Pdb1-3xHA/Lat1-V5/Lpd1-5xFLAG from whole cell extract in a buffer containing 50 mM NaCl with the elution volumes in the SEC via Superose 6 column for every other 500 μl fraction indicated.

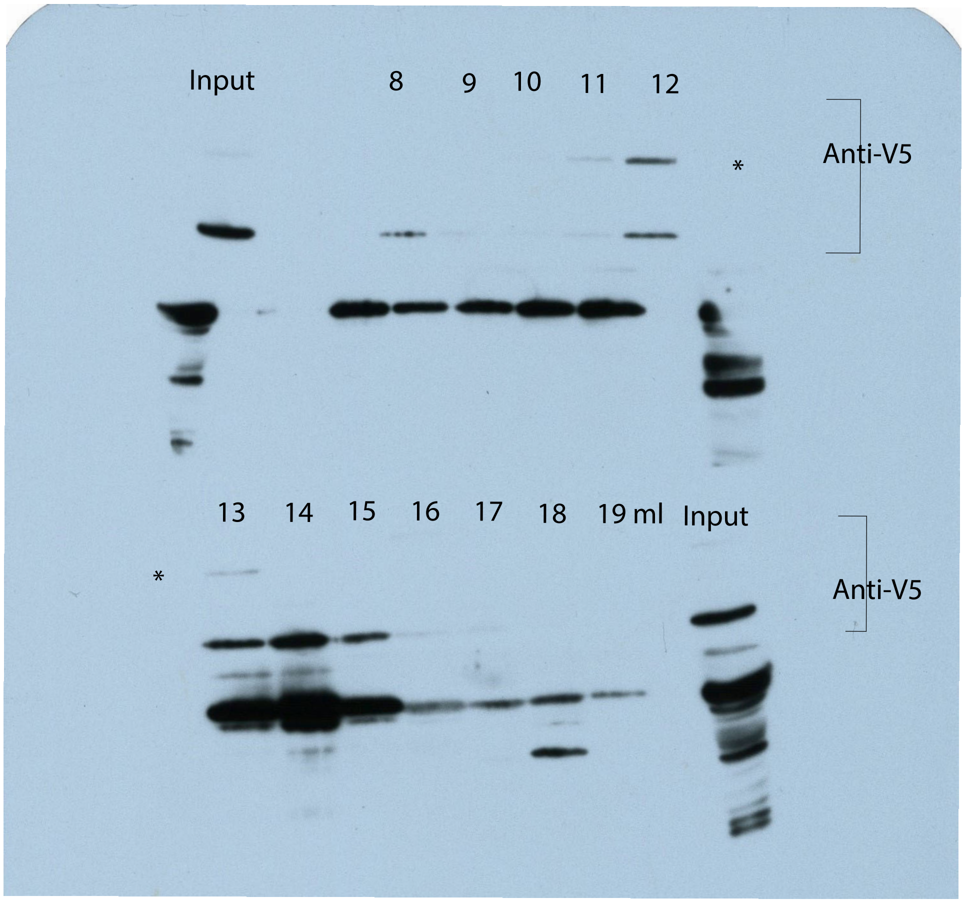

For S2c, Anti-Lamin A and Anti-MTCO2 are shown.
Biological replicates of HS and LS fraction of Pdb1-3xHA/Lat1-V5/Lpd1-5xFLAG were loaded.

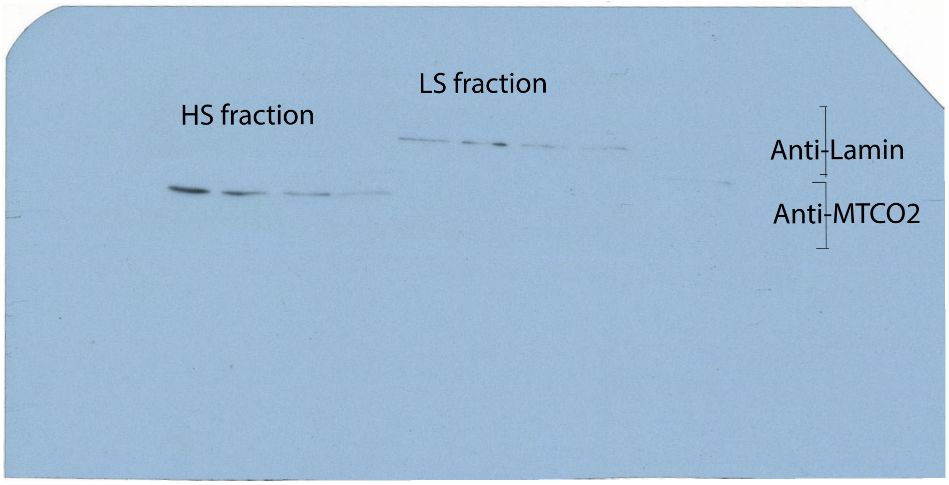

For Fig.S2d-f, Anti-FLAG and Anti-HA blots are shown below.
Size fractions of Pdb1-3xHA/Lat1-V5/Lpd1-5xFLAG from whole cell extract (d), LS fraction (f), and HS fraction (e) in a buffer containing 150 mM NaCl with the elution volumes in the SEC via Superose 6 column for every other 500 µl fraction indicated.

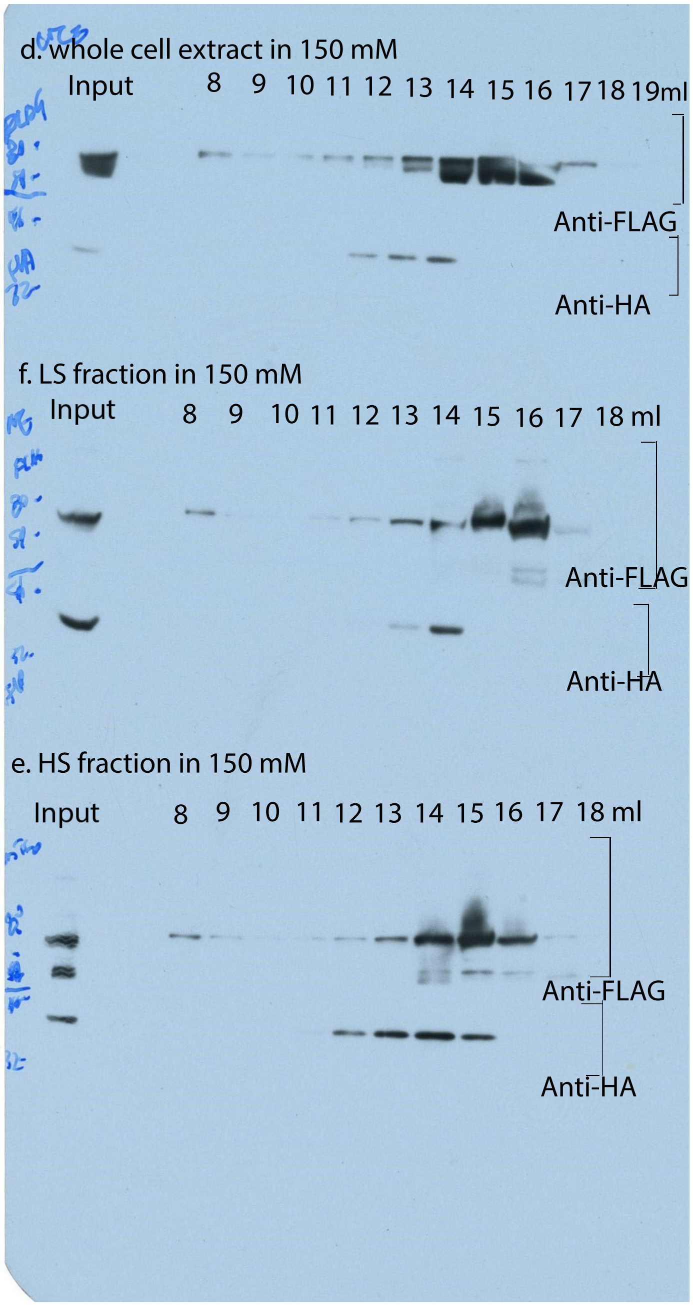

For S4a, Anti-Brg1 blots are shown below.
Size fractions of HEK293T lysates in a buffer containing 350 mM NaCl with the elution volumes in the SEC via Superose 6 column for every other 500 µl fraction indicated.

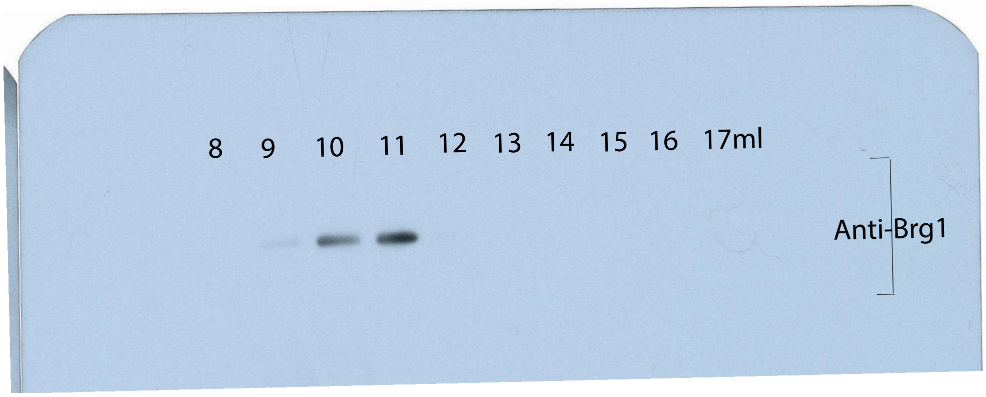

For S4a, Anti-mE1pα blots are shown below.
Size fractions of HEK293T lysates in a buffer containing 350 mM NaCl with the elution volumes in the SEC via Superose 6 column for every other 500 μl fraction indicated.

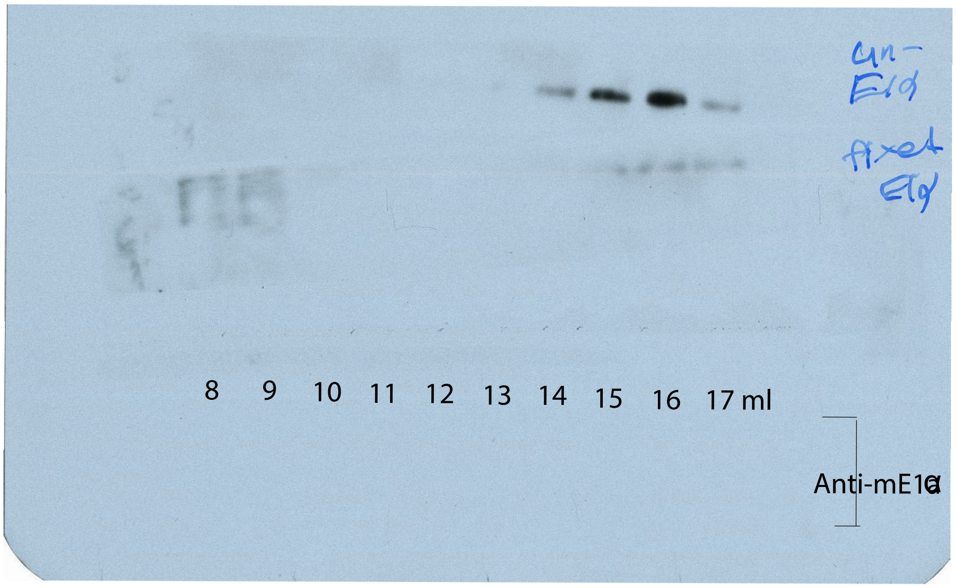

For S4a, Anti-Baf155 blots are shown below.
Size fractions of HEK293T lysates in a buffer containing 350 mM NaCl with the elution volumes in the SEC via Superose 6 column for every other 500 µl fraction indicated.

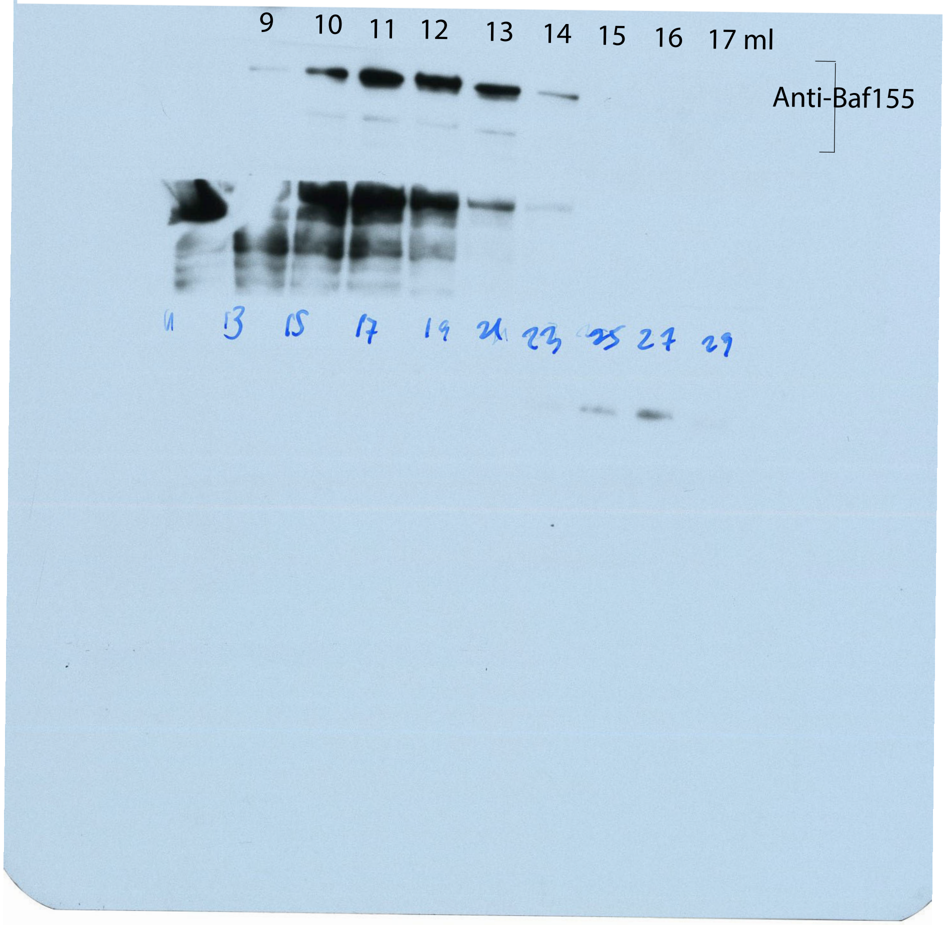

For S6, Anti-mE2p blots are shown.
Size fraction of affinity purified mE1pα (PDHA1-HA) from HEK293T cells in a buffer containing 0 mM NaCl with the Elution volumes in the SEC via Superose 6 column for every 500 μl fraction indicated.

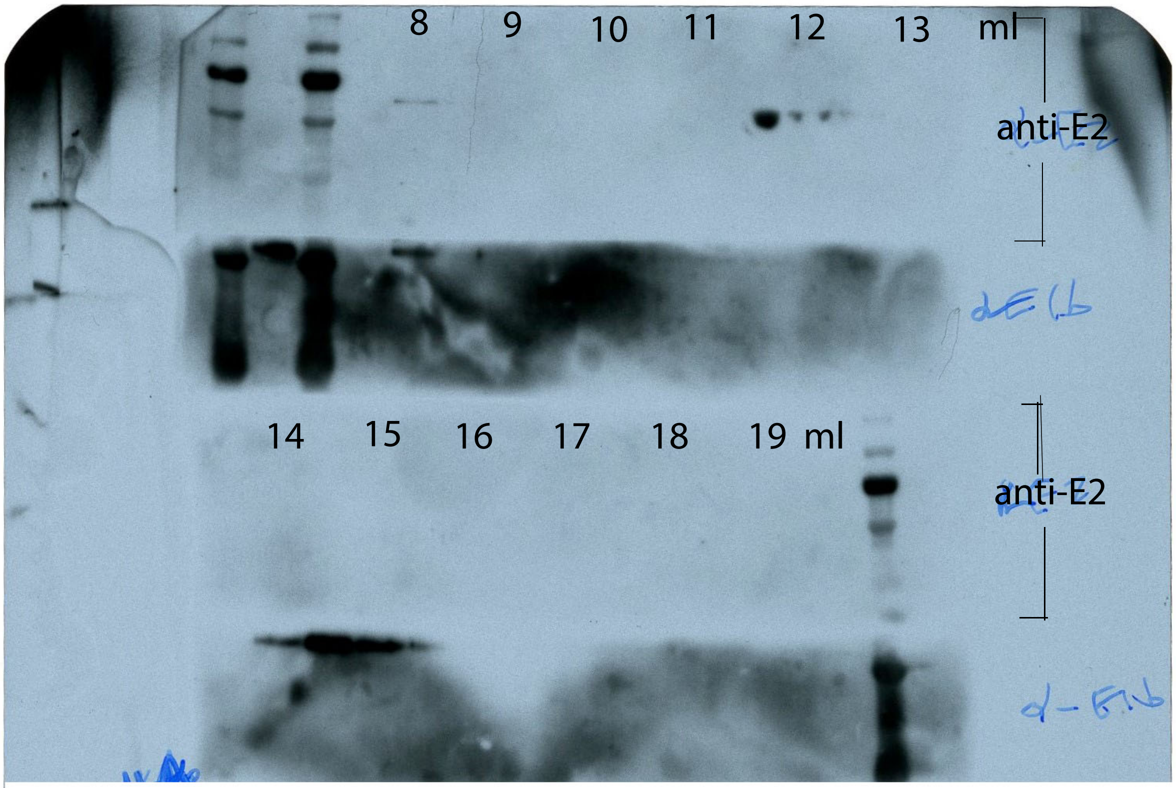

For S6, Anti-HA blots are shown.
Size fraction of affinity purified mE1pα (PDHA1-HA) from HEK293T cells in a buffer containing 0 mM NaCl with the Elution volumes in the SEC via Superose 6 column for every 500 µl fraction indicated.

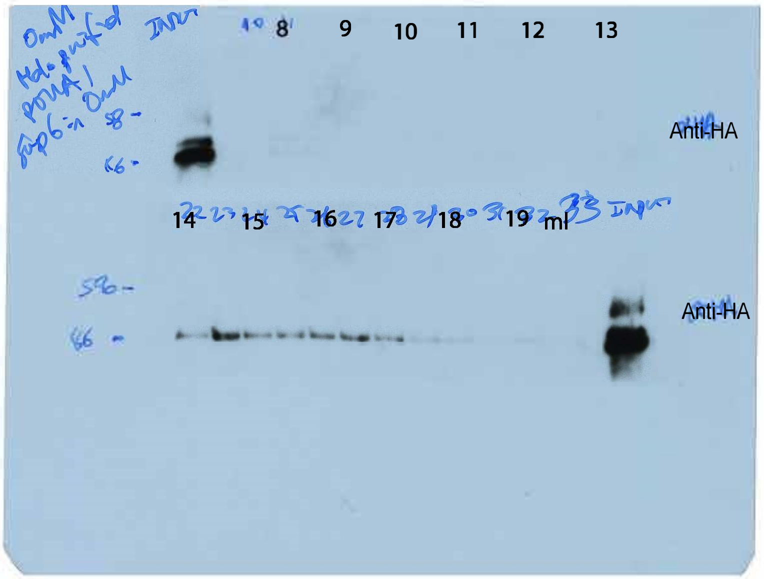

For S7, Anti-FLAG blots are shown.
Same amount of samples containing FLAG purified pkp1/Pdb1-5xFLAG from the catalytic activity assay for 50 mM and 350 mM NaCl conditions was loaded

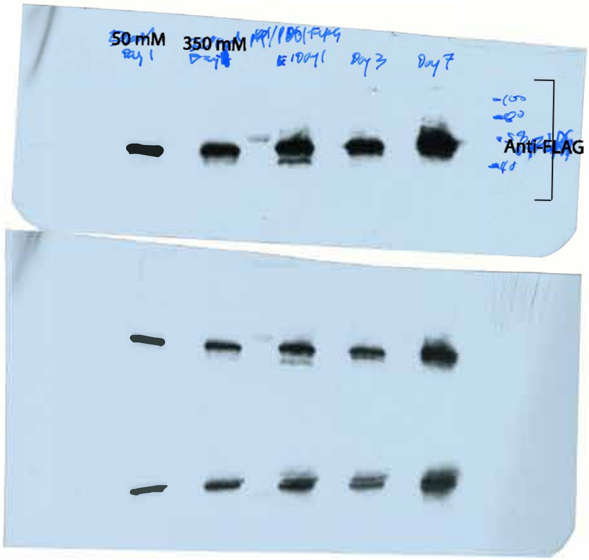

Supplement: S1 Raw images — (PDF) [file pone.0243489.s002.pdf]
